# Economic inequality and academic perseverance: Evidence from 73 countries

**DOI:** 10.1111/bjep.70124

**Published:** 2026-09-15

**Authors:** Yifan Wang, Yanru Liu, Dennis Alonzo

**Affiliations:** ^1^ School of Education University of New South Wales Kensington New South Wales Australia; ^2^ School of Teacher Education Dali University Dali China

**Keywords:** academic perseverance, economic inequality, PISA, socioeconomic status

## Abstract

**Background:**

Academic perseverance is important for educational success, yet research has focused mainly on individual and proximal environmental factors, with limited attention to broader socioeconomic contexts.

**Aims:**

This study examined whether national economic inequality was associated with students' academic perseverance and whether this association varied by family socioeconomic status (SES).

**Samples:**

Data were drawn from four Programme for International Student Assessment cycles conducted in 2000, 2012, 2018 and 2022, comprising 1,374,393 15‐year‐old students from 73 countries.

**Methods:**

Economic inequality was indexed using lagged national Gini coefficients. Country‐level correlations and three‐level weighted multilevel models were estimated, with students nested within schools and countries. Models adjusted for GDP per capita, population, unemployment, gender, age and immigrant background. Cross‐level Gini × SES interactions tested moderation by family SES.

**Results:**

Economic inequality was moderately to strongly positively correlated with country‐level mean academic perseverance across the four waves (*r* = .47–.61). In multilevel models, higher national inequality was also associated with higher individual‐level academic perseverance after adjustment for covariates, explaining approximately 1% of the variance. The Gini × SES interaction was significant in three of the four waves: the positive association was approximately 1.5–1.8 times stronger among lower‐SES students than among higher‐SES students.

**Conclusions:**

Economic inequality was associated with greater academic perseverance, particularly among lower‐SES students. These findings suggest that its educational correlates may be more complex than uniformly negative accounts imply and warrant further research into the underlying mechanisms and longer‐term consequences.

## INTRODUCTION

In recent years, education researchers and practitioners have increasingly recognized perseverance as a critical factor in academic achievement and future success (Waters et al., 2019; Xu et al., 2023; Zhang et al., 2024). As the most central and robust component of the grit construct (Lam & Zhou, 2022), understanding ‘why students persevere in their studies’ has become a question of both theoretical significance and practical economic value. While a body of studies has extensively explored how perseverance is shaped by genetic predispositions (Rimfeld et al., 2016), personality traits (Zhang et al., 2024), family dynamics (Waters et al., 2019), school environments (Buenrostro & Ehrenfeld, 2023) and peer interactions (Clark et al., 2020), distal socioecological factors have often been neglected (Datu, 2021). Reportedly, there are significant variations in perseverance across countries and regions (OECD, 2024; Xu et al., 2023), yet our scientific understanding of this phenomenon remains limited.

One such macroeconomic factor deserving closer attention is economic inequality, which is increasingly recognized as a defining issue of the 21st century. Economic inequality has been extensively linked to negative outcomes in health, wellbeing and social cohesion (Pickett & Wilkinson, 2015; Wilkinson & Pickett, 2009). However, its relationship with students' academic perseverance remains largely unexplored. In this research, we propose and test a hypothesis that economic inequality is associated with students' academic perseverance. We use four waves of large‐scale surveys spanning 22 years and covering 73 countries to investigate this relationship. We further investigate how this relationship is moderated by family socioeconomic status (SES).

This investigation offers several unique perspectives to advance our understanding of academic perseverance. First, it expands the existing theoretical understanding of perseverance, moving from proximal factors such as individual, family and school to more distal socioecological factors. Particularly, given that existing research has demonstrated students' heightened sensitivity to inequality (Mistry et al., 2015; Ruck et al., 2019), investigating this relationship becomes especially necessary. Second, we explore the relationship between perseverance and other economic factors (such as national GDP and family socioeconomic status), extending existing theory. While these economic factors have been previously established as correlates of academic achievement and development, their direct links to perseverance have been relatively underreported. Considering that these economic factors typically correlate highly with inequality, we simultaneously controlled for them in our model. This approach allows us to test the pure association of inequality, rather than variance misattributed to economic levels, while simultaneously examining the relationships between these factors and perseverance. Third, we utilized four waves of PISA data (2022, 2018, 2012, 2000) and applied a lag estimation method, enabling us to capture the lagged relationship between economic inequality and academic perseverance and observe the robustness of this association across a 22‐year time span. Finally, our analysis included representative large‐scale data from 73 countries/regions, which ensures the cross‐cultural universality of our findings and extends insights about perseverance in non‐Western countries.

### Perseverance

Perseverance is defined as an individual's tendency to persist in efforts towards long‐term goals (Duckworth et al., 2007). It is generally viewed as a stable personality trait but exhibits notable domain‐specificity (Schmidt et al., 2019). In educational studies, academic perseverance specifically refers to ‘a student's tendency to complete school work in a timely and thorough manner to the best of one's ability, despite distractions, obstacles, or level of challenge’ (Farrington et al., 2012).

Perseverance is typically placed within the theoretical framework of grit, paired with consistency of interest as its two core components (Duckworth et al., 2007). Despite grit becoming a widely discussed topic, there remains no clear consensus regarding its construct conceptualisation (Ayllón‐Salas et al., 2025; Morell et al., 2021; Muenks et al., 2017). Some studies view perseverance as a relatively independent construct and have observed that perseverance is positively associated with aspirations for higher education (Credé et al., 2017), academic buoyancy (Sudina & Plonsky, 2021), positive academic self‐concept (Yang et al., 2023), intrinsic motivation (Li et al., 2024), mastery orientation (Montano, 2024), behavioural engagement (Waters et al., 2019), and emotional regulation in the face of failure (Feng et al., 2024). A recent meta‐analysis involving 134 studies and 285,331 participants showed that perseverance has a stronger relationship with academic achievement (*r* = .21) than grit (*r* = .19) and consistency of interest (*r* = .08) (Lam & Zhou, 2022). Notably, when controlling for covariates such as conscientiousness, perseverance still significantly explained additional variance in academic achievement, while overall grit and consistency of interest showed almost no incremental validity (Credé et al., 2017). Additionally, some researchers have also raised criticisms that, in practice, consistency of interest is relatively difficult to measure and evaluate at the school level, as school curricula don't allow students to switch interests as freely as they might in hobbies or workplace settings (Schmidt et al., 2019). Considering these situations, in the field of education, studying perseverance as an independent construct appears increasingly feasible and valuable.

Numerous studies have investigated predictors of perseverance at the individual, family and school levels. For example, Rimfeld et al. (2016) found that 37% of the variance in perseverance among 16‐year‐old students can be explained by genetic factors. Sexual minority students who tend towards concealment orientation demonstrate lower perseverance (Goulet & Villatte, 2020). Students who are more curious (von Stumm et al., 2011), hold stronger growth mindsets (Lam & Zhou, 2020), and perceive greater social support (Clark et al., 2020) exhibit higher perseverance.

In family settings, children of parents who practice Strength‐based parenting show greater perseverance (Waters et al., 2019). Children from families with a better atmosphere (Pongpitpitak et al., 2022) and where parents have harmonious emotional relationships (Xia, 2022) demonstrate more perseverance. Notably, students from low SES families exhibit higher levels of perseverance (Li et al., 2023). In school environments, students tend to show more perseverance when teachers employ more autonomy support strategies (Li et al., 2024). Students who receive encouragement focused on challenges and potential display higher perseverance (Tang et al., 2023). Some technology‐based education approaches can influence perseverance in male students (Bi et al., 2024).

### Theoretical framework: Socioecological perspective

Although past research has provided valuable insights into individual and proximal environmental factors related to perseverance, few studies have focused on socio‐ecological factors, particularly macroeconomic factors, which serve as the starting point for our research.

The socioecological perspective, rooted in Bronfenbrenner's (1977) ecological systems theory, posits that human development and behaviour occur within nested environmental systems ranging from immediate interpersonal relationships to broad cultural and economic structures. This framework distinguishes among multiple interconnected levels: from proximal factors (such as family dynamics and school climate) at the microsystem level, to intermediate factors (such as community resources and institutional policies) at the exosystem level, to distal factors (such as cultural values and economic inequality) at the macrosystem level. As scholars have increasingly recognized, individual psychological processes cannot be fully understood in isolation from the broader social and economic contexts in which they are embedded (King et al., 2022; Oishi & Graham, 2010; Ryan et al., 2017; Sommet et al., 2024). In this study, we extend the theoretical framework of perseverance to include economic inequality as an important distal economic factor.

### Economic inequality

Economic inequality refers to the disparities in the distribution of economic resources (for example, income and wealth) within a population (Yang & Konrath, 2023). From 1980 to the present, the income gap between the top and bottom segments within countries has nearly doubled, and the impact of the pandemic has greatly accelerated this process (Chancel et al. 2022). The vast majority of studies link economic inequality to various negative physiological, psychological and social consequences, such as the risk of premature death (Nuru‐Jeter & LaVeist, 2011), depressive disorders (Patel et al., 2018), as well as reduced well‐being (Ngamaba et al., 2018), social trust (Elgar et al., 2020) and intergenerational mobility (Torche, 2015).

These negative aspects of inequality are also widely found in school contexts. Existing research has confirmed that students living in environments with high economic inequality have lower aspirations for continuing education and less peer support (Wilkinson & Pickett, 2009), they are more likely to engage in bullying behaviour or become victims of bullying (Elgar et al., 2019), and feel less sense of belonging at school (King et al., 2022). Additionally, schools in highly unequal regions typically demonstrate poorer classroom discipline (Chiu & Chow, 2011). Finally, countries with high inequality have overall lower levels of academic achievement (Chiu & Chow, 2015), accompanied by larger achievement gaps among students from different SES backgrounds (Chmielewski & Reardon, 2016).

Although most evidence has characterized economic inequality as a substantial problem, emerging research reveals more complex psychological and behavioural outcomes beyond straightforward harm. For instance, Wang et al. (2023) found that low‐SES individuals demonstrate higher self‐improvement motivations when facing inequality manipulation. Corneo and Neher (2014) discovered that unequal environments shape higher work ethics and engagement, which cannot be explained solely by economic needs. In school settings, Sommet et al. (2023) found that economic inequality stimulates instrumental cooperation awareness among middle school students, which is typically associated with higher academic achievement. Furthermore, within another theoretical framework, they suggest that inequality may elicit heightened effort, pride and task interest in students—though the mechanisms and implications of these patterns remain subjects of ongoing debate (Sommet et al., 2024).

These complex patterns of response to inequality warrant further investigation. However, these findings do not suggest that inequality produces beneficial outcomes. Rather, they may reflect individuals' capacities for adaptation and self‐improvement in unequal environments, particularly among disadvantaged groups with low SES (Wang et al., 2023). These perspectives have received limited attention in prior research.

### Inequality and perseverance in economic perspective

Several lines of evidence have already potentially linked economic inequality and perseverance, particularly in economics and organizational psychology. We first briefly review these findings related to adults and examine how much they can be applied to school environments.

First, economic inequality promotes a tendency toward hard work, which is usually an important aspect of perseverance. Lazear and Rosen's tournament theory suggests that unequal economic environments shape incentive structures that encourage employees to exert more effort to climb higher on the salary ladder (Lazear & Rosen, 1981). Supporting this argument, numerous studies across different independent groups have found that inequality is associated with longer working hours (Alexiou & Kartiyasa, 2020; Bell & Freeman, 2001; Bowles & Park, 2005). Furthermore, Corneo and Neher (2014) conducted a 30‐year survey across 34 countries and found that higher economic inequality correlates with people more strongly believing that work should be viewed as the first priority, taking job searches more seriously and feeling that work is more of an expression of social responsibility. Notably, these tendencies towards harder work often appear alongside impaired perceptions of fairness, perceived intergenerational mobility, job satisfaction and heightened social comparison (Ahn et al., 2016; Cheung & Lucas, 2016; Reyes & Gasparini, 2022). In other words, the hard work that results from inequality is often uncomfortable or discouraging, with less pleasure, which corresponds to another aspect of perseverance: persistence and tolerance. Some evidence also supports this point, namely that economic inequality is associated with workers having higher tolerance for work and being less selective, especially when they find jobs with lower pay or unpleasant conditions (Corneo & Neher, 2014).

### Inequality and perseverance in school

Although the relationship between inequality and perseverance in school settings has not been empirically established, three arguments support our hypothesis that inequality is positively associated with academic perseverance: family environment, competitiveness and the pursuit of wealth and status.

First, considering that inequality promotes perseverance among adults in work environments, there is reason to believe that this relationship may be transmitted through family education. Evidence has shown that parents in more unequal regions not only work harder themselves but are also more inclined to teach their children that hard work is an important life value (Corneo & Neher, 2014; Doepke et al., 2019). Moreover, given that economic inequality potentially increases the economic returns of education for families across all social strata (i.e., the economic risks/benefits of falling from upper classes and rising from lower classes become greater), parents are more likely to be motivated to encourage or even pressure their children to achieve educational success (Doepke et al., 2019). For example, parents in unequal regions may be more inclined to employ authoritarian and reinforcement‐based parenting strategies (Schneider & Schenck‐Fontaine, 2022), and increase financial investment in their children's education (Schneider et al., 2018). This may lead students to demonstrate greater perseverance in their studies due to parental expectations and family pressure.

Second, the increased competitiveness brought about by economic inequality may drive students towards greater perseverance in learning. In environments of economic inequality, the large disparities in resource allocation foster a highly competitive atmosphere (Sommet et al., 2019; Sommet & Elliot, 2023). This phenomenon is also reflected in school environments; for example, Sommet found that inequality stimulates both a more competitive learning atmosphere among students and enhances competitive mindsets (Sommet et al., 2023). According to achievement goal theory (Elliot & McGregor, 2001; Murayama & Elliot, 2012), when students adopt approach‐oriented competitive motivations, they may view academic challenges and competition as opportunities to enhance their status, invest more time in academics and demonstrate stronger persistence in the face of difficulties. Some empirical studies also support this hypothesis; for instance, Dugan et al. (2019) found a moderate correlation between perseverance and competitive awareness (*r* = .35). Considering these results, perseverance may serve as a behavioural outcome in response to the high level of competition brought about by inequality.

Third, economic inequality may increase perseverance via fueling the pursuit of wealth and status. Numerous studies have demonstrated that higher economic inequality heightens individuals' desire for status and wealth, both in adults and students (Wang et al., 2023). In more unequal regions, people consider wealth and status more important (Walasek & Brown, 2019), and they tend to achieve a positive identity through acquiring wealth, along with categorizing others (Jetten et al., 2021). Given these considerations, students may view the potential future wealth and status as a long‐term goal. Moreover, from the sociological perspective, educational systems take an important responsibility for occupational readiness and selection (Darnon et al., 2009). That is, not only should school be seen as a place of academic development, but it may also become the social sorting machines that gatekeep access to highly compensated and high‐status occupations (Domina et al., 2017). In unequal environments, education is an effective pathway for personal investment and intergenerational mobility (Cheng & Wen, 2019). Therefore, students may become more aware of the importance of perseverance and view it as a crucial means to succeed in high‐stakes tests and achieve future economic success and status.

### Family socioeconomic status as a moderator

Although we believe that economic inequality may enhance students' academic perseverance, the actual situation may be quite complex. In particular, the magnitude and direction of this association likely vary substantially across different social backgrounds. Identifying potential moderators is crucial as it helps us understand how inequality operates across different social strata, particularly for disadvantaged students (King et al., 2022).

One important moderator of the inequality association might be Family SES, which reflects the family's ability to obtain material and social resources (Matthews & Gallo, 2011). Operationally, it is typically defined as a composite index combining parents' highest occupational status, parents' highest educational level, and home possessions (Chen et al., 2025; Wang et al., 2025; Zhang et al., 2023). We hypothesize that students from low SES families will demonstrate greater academic perseverance when facing economic inequality, based on two reasons.

First, low SES families typically have fewer material resources, which exposes them to greater economic pressure and life challenges when facing environments of economic inequality. For instance, they may need to accumulate sufficient retirement funds and emergency savings to compensate for limited social security systems (Brady & Bostic, 2015); higher healthcare costs to address poorer physical and mental health outcomes (Rambotti, 2015); and more investment in transportation and commuting, as affordable housing is typically located far from employment centres and quality schools (Ludwig et al., 2012). These objective economic challenges may motivate students from low SES families to demonstrate perseverance in their studies to overcome existing life challenges and attempt to obtain better returns through education (whether voluntarily or influenced by parental values). In contrast, high–SES families possess more abundant material resources as a protective shield, making them less affected by the negative consequences of economic inequality. As a result, students from these families may not view academic perseverance as a necessary pathway to overcome economic disadvantages.

Second, lower‐SES families typically exhibit a stronger status competition mindset (Wang et al., 2023). Research indicates that low–SES individuals often experience greater status anxiety in unequal environments, and more actively seek to improve their social position to regain a sense of control within an unequal social context (Pickett & Wilkinson, 2015; Wang et al., 2023). Furthermore, low SES individuals typically demonstrate a stronger tendency to ‘surpass others’, which aligns with the characteristics of approach goals in achievement goal theory (Elliot & McGregor, 2001; Loughnan et al., 2011). Research has confirmed that individuals with an approach goal orientation display greater perseverance and effort when facing difficulties, and are willing to invest more time in their endeavours (Alhadabi & Karpinski, 2020; Murayama & Elliot, 2012). Therefore, it is reasonable to infer that economic inequality may encourage low SES students to exhibit stronger academic perseverance to satisfy their more intense status needs and social comparison motives. In contrast, high SES individuals tend to experience more downward social comparison anxiety: they worry about sliding from their existing social position or being surpassed by others (Jetten et al., 2021; Mols & Jetten, 2017). In learning environments, this mindset resembles avoidance motivation, which has been proven to be associated with earlier abandonment of tasks and lower perseverance (Elliot & McGregor, 2001; Sideridis & Kaplan, 2011).

### Overview

In this study, we conducted four pre‐registered studies to test the following hypotheses:

*At the country level, economic inequality is positively associated with academic perseverance at the national level*.

*Country‐level economic inequality is positively associated with individual students' academic perseverance*.

*Family socioeconomic status moderates the cross‐level association between country‐level economic inequality and individual students' academic perseverance, such that this association is stronger among students from low‐SES families*.

To test the above hypotheses, we analysed four waves of large‐scale cross‐national data from the OECD Program for International Student Assessment (PISA) 2000, 2012, 2018 and 2022, which allowed us to include results from 73 countries worldwide, spanning 22 years and a total of 1,374,393 students aged 15. We used all four waves of data to test [Link], [Link], [Link].

We adopted several improved methodological approaches to better support our proposed hypotheses, which are related to concerns in the field of economic inequality. First, a substantial body of research has demonstrated that confounding factors, particularly absolute wealth levels, can significantly interfere with estimating the effects of inequality. As King et al. (2022) pointed out, some studies have found that when different covariates are considered, the relationship between inequality and educational outcomes may disappear. Consistent with previous research, in this study, we controlled for covariates such as GDP, unemployment rates, age and immigration background (Sommet et al., 2023). This allowed us to estimate the association between inequality and academic perseverance relatively independent of individual factors and societal wealth levels.

Second, considering that the effects associated with income inequality may be relatively small, we incorporated as many PISA waves as possible. This allowed us to gather information from more countries and larger samples of participants to capture small effect sizes. Additionally, analyses across different waves enabled us to identify differences between various time points, measurement methods and countries measured, and to test whether results could be replicated.

This research has been approved by the Ethics Committee of the University of New South Wales (iRECS 8431). We registered the complete research process on the Open Science Framework (OSF) platform, and the analysis process is a priori (unless otherwise specified). Supplementary materials include (a) detailed participant information, (b) covariate collection procedures, (c) robustness checks for alternative model specifications, (d) sensitivity analyses for missing data and (e) power analysis procedures. The preregistration report, dataset and analysis code can be found on the OSF (https://osf.io/xfm9q/?view_only=21bd84bb5ce14733a6810f01c5937b0e).

## METHODS

### Participants

We used data from PISA 2022 (Study A), 2018 (Study B), 2012 (Study C) and 2000 (Study D) to test our hypotheses. Wave selection was based on the availability of perseverance items. Only these four cycles included such measures, and the remaining waves (2003, 2006, 2009 and 2015) were therefore excluded. PISA aims to evaluate and measure the mathematics, reading, and science competencies and literacy of 15‐year‐old students across the globe. It also collects extensive indicators on student demographics, lifestyle, psychological well‐being and family and school environments. As specified in the preregistration, we included all countries with available Gini coefficients (79.2%) and participants with complete perseverance data (72.3%), resulting in a final sample of 1,374,393 students from 73 countries (PISA 2000: 52,510 students in 22 countries; PISA 2012: 480,174 students in 52 countries; PISA 2018: 494,559 students in 72 countries; PISA 2022: 347,150 students in 44 countries). These sample sizes allow us to detect small effect sizes (*f* ^2^ = .02, *α* = .05, power = .99). The distributions of perseverance scores in the analytic and full PISA samples are presented in Figure S2.

### Measures

#### Economic inequality

National‐level economic inequality was measured using the Gini coefficient provided by the World Bank Open Data (https://data.worldbank.org). The Gini coefficient ranges from 0 (absolute equality where everyone has the same wealth) to 100 (absolute inequality where all wealth is concentrated in a single individual). Following Chua et al. (2019), we employed a lagged estimation approach. This temporal separation helps to reduce simultaneity bias by ensuring that the independent variable is measured before the dependent variable. Accordingly, we selected the Gini coefficients from 2020, 2016, 2010 and 1998 as key indicators for analysis. In cases where data for a given year were missing, we used the closest available estimate from a previous year as a substitute. In this research, the Gini coefficient ranged from 23 (Denmark in 1998) to 59.6 (Brazil in 1998), *M* (*SD*) = 34.63 (6.83).

#### Perseverance

PISA 2022 (Study A), PISA 2018 (Study B), PISA 2012 (Study C) and PISA 2000 (Study D) each employed different methods to measure perseverance (Tables S5 and S6).

In Study A, we used the PERSEVAGR score from PISA 2022, a WLE index assessing perseverance based on students' responses to 10 items (OECD, 2024). Each item is rated on a scale from 1 (Strongly disagree) to 5 (Strongly agree). Example items include ‘I keep working on a task until it is finished’ and ‘I finish tasks that I started even when they become boring’. *M* (*SD*) = .006 (.993), *α* = .77.

For Study B, we used the mean score of the four perseverance items from PISA 2018 (1 = Strongly disagree, 4 = Strongly agree) (Montano, 2024). Examples of measurements include ‘Once I start a task, I persist until it is finished’ and ‘If I am not good at something, I would rather keep struggling to master it than move on to something I may be good at’. *M* (*SD*) = 3.03 (.60), *α* = .93.

For Study C, we used the PERSEV score from PISA 2012, a WLE index assessing perseverance based on students' responses to 5 items (OECD, 2014). Each item is rated on a scale from 1 (Not at all like me) to 5 (Very much like me). Example items include ‘I continue working on tasks until everything is perfect’ and ‘When confronted with a problem, I do more than what is expected of me’. *M* (*SD*) = .137 (.995), *α* = .78.

In Study D, we utilized the EFFPER score from PISA 2000, a WLE index that measures academic effort and perseverance (Adams & Wu, 2003). Example items include ‘I will persist in studying even if the material is difficult’ and ‘I will try my best to learn the knowledge and skills being taught’. This scale is similar to traditional perseverance measures, such as the Short Grit Scale (Duckworth et al., 2007) (e.g., ‘I am a hard worker’) and the Values in Action Strength Survey (Peterson & Seligman, 2004) (e.g., ‘Whenever I do something, I put all my effort into it’). Responses were rated on a scale from 1 (almost never) to 4 (almost always), *M* (*SD*) = .09 (1.00), *α* = .77.

#### Socioeconomic status as moderator

For all four studies, we used the index of Economic, Social and Cultural Status (ESCS) score as the measure of SES. ESCS is an officially constructed WLE composite index by the OECD, integrating three key dimensions: parental occupational status, parental educational attainment and household possessions. Compared to other single PISA indicators, it provides a more comprehensive assessment of students' family socioeconomic background (Avvisati, 2020). The index is standardized to ensure comparability across PISA cycles, making it well‐suited for the cross‐wave design of the present study. ESCS has also been widely adopted in large‐scale cross‐national studies of a similar nature (Avvisati, 2020; King et al., 2022).

#### Country‐level covariates

The following national‐level control variables were included: (a) population of the country, (b) unemployment rate and (c) gross domestic product (GDP) per capita. Similar to the Gini coefficient, all data were sourced from World Bank Open Data and applied using a lag estimation. If data for a given year were unavailable, we used the closest available estimate from a preceding year.

#### Individual‐level covariates

The following individual‐level control variables were included: (a) gender, (b) age and (c) immigrant background status. All data were obtained from self‐reports by PISA participants (Table S1). The rationale for selecting each covariate is detailed below.

First, for gender, research indicates that women perceive economic inequality more strongly than men (Chen et al., 2024; Zamberlan et al., 2025), and that higher levels of economic inequality reinforce competitive normative climates that further constrain women's access to opportunities and resources (Moreno‐Bella et al., 2022). Additionally, several empirical studies have found that girls demonstrate significantly higher levels of grit and perseverance than boys (Kannangara et al., 2018; Oriol et al., 2017). Given that gender is potentially associated with both perceived economic inequality and perseverance, it was included as a control variable in the analyses.

Second, regarding age, Credé et al. (2017), in a meta‐analytic synthesis of the grit literature encompassing 584 effect sizes from 88 independent samples (*N* = 66,807), established perseverance of effort as the core facet of grit and confirmed a positive association between grit and age. Shubert et al. (2020), using an accelerated longitudinal design with 4055 adolescents in Grades 6–12, further demonstrated that perseverance followed a growth trajectory in early‐to‐middle adolescence that levelled off in later adolescence. Together, these findings suggest that perseverance is developmentally sensitive during adolescence. Given that even modest age variation within the PISA sample may introduce systematic variance in perseverance scores, age was included as a covariate in the analyses.

Third, for immigrant background, socioeconomic disadvantage is considerably more prevalent among immigrant students than among native students in most participating countries (OECD, 2023), suggesting that immigrant background may be associated with greater objective exposure to economic inequality. At the same time, a number of studies tentatively suggest that immigrant status may be linked to stronger academic perseverance. Fuligni (1997), drawing on a sample of approximately 1100 adolescents from Latino, East Asian, Filipino and European backgrounds, found that first‐ and second‐generation immigrant students outperformed their native peers in mathematics and English, an advantage attributable in part to more positive academic attitudes and higher behavioural engagement. Chiu et al. (2012), in a multilevel analysis of 276,165 15‐year‐olds across 41 countries, similarly found that immigrant students displayed more positive attitudes toward school than their native counterparts. Given that cognitive engagement with school overlaps conceptually with perseverance to some degree (Fredricks et al., 2004), these patterns may indirectly suggest a tendency toward greater sustained effort among immigrant students when facing academic challenges. In light of these considerations, immigrant background was included as a control variable in the analyses.

### Pre‐registered analysis

All analyses were conducted using R 4.3.3 (R Core Team, 2024), with statistical significance set at *p* = .05 (two‐tailed). Prior to formal analysis, all variables were centred to ensure that the intercept within the models fell within the range of the perseverance measure.

We constructed a three‐level multilevel linear model (MLM) to examine the association between country‐level economic inequality and individual students' academic perseverance, accounting for the nested structure of students (Level 1) within schools (Level 2) within countries (Level 3). This approach is consistent with established practice in cross‐national research on inequality and educational outcomes (King et al., 2022; Sommet et al., 2023). Prior to constructing the full model, we estimated a null model for each study to compute intraclass correlations (ICCs), confirming meaningful variance at both the country level (L3 ICC: 2.38%, 5.65%, 6.96% and 4.77% for Studies A–D, respectively) and the school level (L2 ICC: 1.50%, 3.07%, 1.33% and 2.96%, respectively), supporting the use of a three‐level structure.

At Level 3, the Gini coefficient was entered as the primary predictor, with GDP per capita, national population and unemployment rate as country‐level covariates. Schools were modelled with a random intercept. At Level 1, academic perseverance served as the outcome variable, with gender, age and immigrant background included as covariates. Family SES was treated as an individual‐level moderator, group‐mean centred within countries to separate individual‐level from country‐level SES effects, and entered into a cross‐level interaction with the Gini coefficient at Level 3. To appropriately model this interaction, we allowed the slope of SES on perseverance to vary randomly across countries.

As a robustness check, we employed ordinary least squares (OLS) with robust standard errors and cluster options for countries as a supplementary analysis. The model regressed academic perseverance on all individual‐level covariates (gender, age and immigrant background) and country‐level covariates (GDP, unemployment rate and national population) as fixed effects, with Huber‐White robust standard errors clustered at the country level. To examine moderation by family SES, we further included the product term of SES and the Gini coefficient as a fixed effect in this OLS model (Lee et al., 2023). Compared to conventional multilevel models, this approach does not impose the assumption that random effects are independent of covariates (Lee et al., 2023; Raudenbush & Bryk, 2002). This is particularly relevant here, as national‐level covariates such as GDP and unemployment rate are likely correlated with country‐level random intercepts, and imposing this assumption in multilevel models could introduce bias in parameter estimates. We acknowledge that this model does not separately estimate school‐level variance, and therefore present it as a supplementary analysis.

To ensure that missing data did not systematically affect the results, we employed multilevel multiple imputation based on the Markov Chain Monte Carlo (MCMC) algorithm (Grund et al., 2016). Compared to traditional multiple imputation methods, this approach better accounts for the interdependence between missing data patterns and the hierarchical structure of the data (Grund et al., 2016). Using the mitml package, we conducted 50,000 burn‐in iterations followed by 5000 formal iterations to achieve stable estimates, ultimately generating 100 imputed datasets. Robustness checks indicated that the imputed datasets produced results nearly identical to those obtained with the original data (Table S2).

### Additional non‐preregistered analyses

In the revised primary analyses, three‐level weighted linear mixed‐effects models were estimated using the WeMix package in R. The PISA final student sampling weight was applied at Level 1, and the corresponding school sampling weight was applied at Level 2. Because participating countries were not selected through a common probability‐sampling design, no probability‐based sampling weight was available at the country level, and no additional weighting was applied at Level 3.

We also conducted several supplementary analyses that were not specified in the preregistration. First, we examined the bivariate association between the Gini coefficient and country‐level mean academic perseverance and conducted country‐level multiple regression analyses adjusting for GDP per capita, population size and unemployment. We further conducted leave‐one‐country‐out analyses, analyses excluding countries in the Latin American and Caribbean region, and models incorporating world‐region fixed effects to assess whether the country‐level association was driven by individual influential countries or regional clustering (Table S15).

Second, we conducted sensitivity analyses to examine potential cultural confounding and cross‐national differences in response styles. Because the availability of cultural indicators differed across countries and PISA waves, Long‐Term Orientation, Individualism, Uncertainty Avoidance and cultural tightness were separately included as country‐level covariates (Tables S8–S11). We also constructed country‐level indices of extreme response style and acquiescent response style using questionnaire items that were conceptually distinct from the academic perseverance measures and separately included these indices in the multilevel models (Tables S12–S14).

Third, we examined whether the findings depended on the temporal specification of the Gini coefficient. The revised models were re‐estimated using contemporaneous Gini values, annual lags ranging from 1 to 10 years, and averages over the preceding 1–3, 1–5 and 1–10 years. In each analysis, the country‐level covariates were defined using the corresponding temporal specification (Table S16).

Fourth, we examined whether items that might reflect consistency of interest rather than perseverance of effort affected the findings. These items were removed from the PISA 2012 and PISA 2018 measures, academic perseverance scores were recomputed, and the analyses were repeated using the revised measures (Table S5 and Figure S1).

Finally, following the reviewer's suggestion, we estimated a hybrid (Mundlak) multilevel model using the pooled data from the four repeated cross‐sectional PISA waves to distinguish the between‐country component of economic inequality from its within‐country, across‐wave component. Because different measures of academic perseverance were used across the PISA waves, perseverance scores were standardized separately within each wave before pooling the data. The model simultaneously included each country's across‐wave mean Gini coefficient and the country‐mean‐centred Gini coefficient, together with the corresponding between‐country and within‐country, across‐wave components of the country‐level covariates (Table S7). Importantly, the within‐country component in this analysis captures changes within the same country across PISA waves rather than longitudinal changes within the same individuals.

## RESULTS

### Inequality nations, persevering countries

We first examined the association between national Gini coefficients and country‐level perseverance to test H1. As hypothesized, the Pearson correlation between economic inequality and national‐level means of academic perseverance shows moderate to strong associations, Study A: *r* = .61, *p* < .001, 95% CI = [.387, .770]; Study B: *r* = .47, *p* < .001, 95% CI = [.267, .632]; Study C: *r* = .48, *p* < .001, 95% CI = [.232, .662]; Study D: *r* = .49, *p* = .020, 95% CI = [.087, .756] (Figure 1). These associations remained statistically significant after adjusting for GDP per capita, population size and unemployment rate in country‐level multiple regression analyses (Table S4). These findings consistently indicate that higher economic inequality is associated with greater academic perseverance at the national level.

**FIGURE 1:**
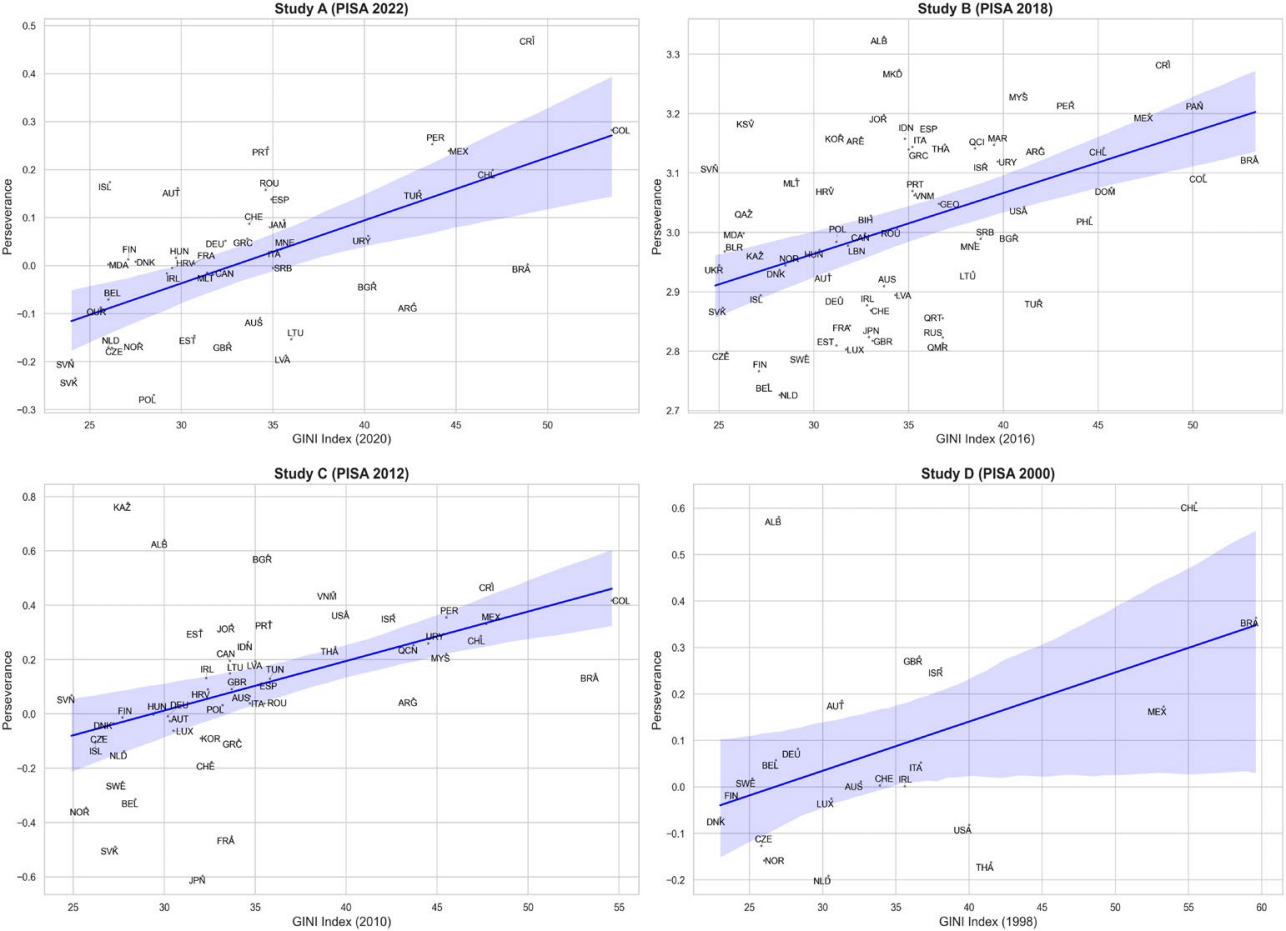
Economic inequality predicts higher academic perseverance at the national level. Scatterplots show the relationship between Gini Index (economic inequality) and standardized academic perseverance scores across countries in four PISA cycles: (A) PISA 2022 with Gini 2020, (B) PISA 2018 with Gini 2016, (C) PISA 2012 with Gini 2010 and (D) PISA 2000 with Gini 1998. Each point represents a country. Blue lines show linear regression fits with 95% confidence intervals (shaded areas).

### Inequality contexts, persevering students

Furthermore, we employed a 3‐level nested MLM to examine the relationship between national Gini coefficients and individual‐level perseverance, providing preliminary verification of H2 (Table 1). Across all four waves of PISA data, economic inequality from 2 years prior was significantly associated with students' subsequent higher academic perseverance. It alone explained .8%–1.9% of the variance (*R* ^2^). After controlling for national economic conditions and demographic variables, this association remained significant with minimal change in coefficient magnitude (Table 1). This finding was consistent across both the OLS model with clustered standard errors and the multilevel model (Table S3).

**TABLE 1:** Associations between economic inequality and academic perseverance across PISA 2000, 2012, 2018 and 2022.

Measure	Study A		Study B		Study C		Study D	
	(PISA 2022)		(PISA 2018)		(PISA 2012)		(PISA 2000)	
Number of students/schools/countries	347,150/13,800/44		494,559/20,297/72		480,174/17,865/52		52,510/2797/22	
	***B***	**95% CI**	***B***	**95% CI**	***B***	**95% CI**	***B***	**95% CI**
Model without control								
Gini coefficient	.013***	[.008, .018]	.010***	[.006, .015]	.018***	[.009, .028]	.010**	[.002, .019]
Intercept	.016	[−.021, .053]	3.011***	[2.981, 3.041]	.134***	[.066, .203]	.084*	[.002, .166]
Country residual variance	.016	[.010, .023]	.045	[.032, .062]	.061	[.041, .089]	.038	[.020, .067]
School‐level residual variance	.015	[.013, .016]	.031	[.029, .032]	.013	[.012, .015]	.03	[.025, .034]
Student‐level residual variance	.963	[.958, .968]	.917	[.914, .921]	.927	[.922, .932]	.928	[.916, .939]
*R* ^2^	.008		.011		.019		.011	
Model with control								
Gini coefficient	.009*	[.001, .018]	.007**	[.002, .012]	.014**	[.004, .025]	.020***	[.011, .030]
SES (country‐mean)	−.232*	[−.430, −.034]	−.086	[−.174, .003]	.072	[−.130, .274]	.243	[−.018, .503]
SES (within‐country)	.104***	[.090, .119]	.049***	[.042, .055]	.109***	[.092, .125]	.093***	[.059, .128]
Age	.039***	[.027, .051]	−.033***	[−.039, −.027]	.057***	[.044, .070]	.007***	[.004, .010]
Gender (+.5 = Boys)	.088***	[.081, .095]	−.072***	[−.075, −.068]	.025***	[.017, .032]	−.218***	[−.235, −.200]
Origin (+.5 = Immigrant)	.062***	[.047, .076]	.048***	[.040, .055]	−.121***	[−.145, −.097]	.110***	[.069, .151]
GDP	.182*	[.003, .362]	−.047	[−.144, .050]	−.403**	[−.646, −.160]	−.296	[−.597, .006]
National population	−.058	[−.127, .010]	−.023	[−.065, .019]	.316***	[.056, .576]	−.134*	[−.251, −.018]
Unemployment rate	.002	[−.010, .014]	.004	[−.002, .010]	−.006	[−.020, .008]	.025*	[.006, .045]
Gini × SES	−.003***	[−.005, −.001]	−.001**	[−.002, −.001]	−.003*	[−.005, −.001]	−.002	[−.005, .001]
Intercept	−.012	[−.059, .035]	2.989***	[2.953, 3.025]	.106*	[.025, .186]	.117**	[.053, .181]
Country residual variance	.012	[.009, .022]	.041	[.028, .054]	.042	[.026, .057]	.018	[.008, .028]
School‐level residual variance	.013	[.011, .016]	.027	[.025, .028]	.011	[.010, .013]	.028	[.024, .033]
Student‐level residual variance	.945	[.940, .949]	.901	[.897, .904]	.914	[.909, .919]	.909	[.898, .920]
*R* ^2^	.018		.031		.044		.047	

*Note*: All multilevel models were estimated at the student level and incorporated the PISA final student and school sampling weight.

Abbreviations: CI, confidence interval; GDP, gross domestic product per capita; SES, socioeconomic status.

* *p* < .05.

** *p* < .01.

*** *p* < .001.

### Family SES as a moderator

Finally, we tested whether the association between economic inequality and academic perseverance varied across family SES levels using cross‐level interaction analyses. The Gini × SES interaction was statistically significant in Studies A–C but not in Study D (Table 1).

In Studies A–C, the positive association between economic inequality and academic perseverance was stronger among students from lower‐SES backgrounds and comparatively weaker among students from higher‐SES backgrounds. Simple‐slope analyses showed that the slopes for low‐SES students were .13, .06 and .14 in Studies A, B and C, respectively, compared with .08, .04 and .08 for high‐SES students (Figure 2). Thus, the estimated association was approximately 1.5 to 1.8 times stronger among low‐SES students than among high‐SES students. Overall, H3 was supported in three of the four PISA waves.

**FIGURE 2:**
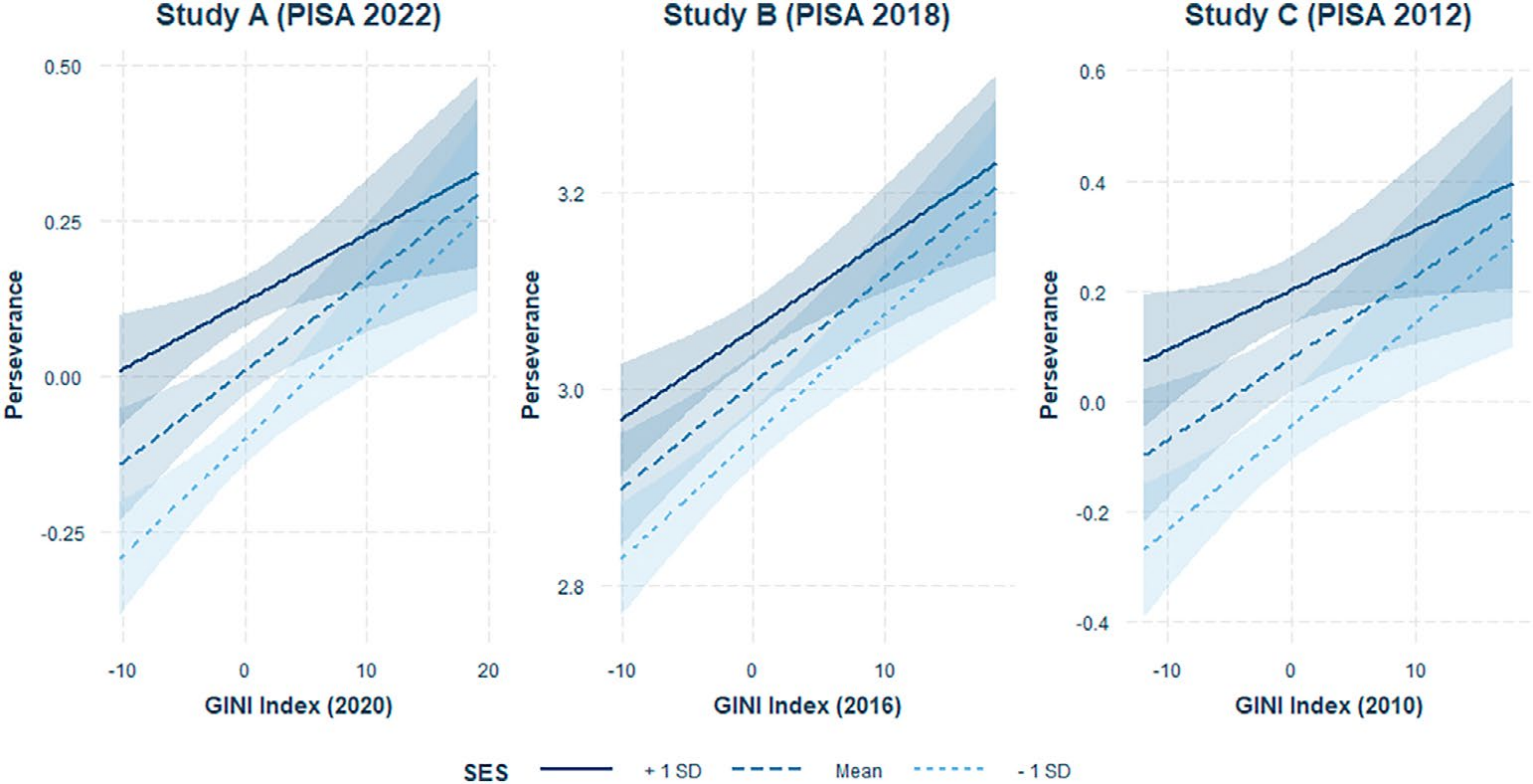
SES moderates the relationship between economic inequality and academic perseverance. Lines represent predicted values at different socioeconomic status (SES) levels: +1 *SD* (solid), mean (dashed) and −1 *SD* (dotted). Shaded areas indicate 95% confidence intervals.

## DISCUSSION

The results from the four studies provide a picture of economic inequality and perseverance in school settings. Through four waves of worldwide student surveys in 73 countries, national economic inequality was robustly and positively associated with students' academic perseverance. Moreover, family SES significantly moderated this relationship: the association between inequality and perseverance was 1.5–1.8 times stronger among low‐SES students compared to their high‐SES counterparts. These findings are broadly consistent with previous research in adult samples reporting positive associations between economic inequality and work‐related effort or engagement (Alexiou & Kartiyasa, 2020; Corneo & Neher, 2014).

While previous studies have explored the relationship between individual, family and school factors and academic perseverance, broader socioeconomic factors remain underexplored (Datu, 2021). Our work expands this critical area of enquiry by focusing on economic inequality. Moderate to strong correlations (*r* = .47–.61) exist between inequality and academic perseverance at the national level. This effect size is similar to past research, such as King et al. (2022) who reported a large effect size between economic inequality and school belongingness (*r* = .89), and Pickett and Wilkinson (2015) who reported a moderate effect size between inequality and mathematics achievement (*r* = −.50). Taken together, this evidence suggests that economic inequality is meaningfully associated with between‐country variation in educational outcomes. As Sommet et al. (2023) noted: ‘there is no reason to believe that the effects of inequality stop at the school gates (p.247).’ Future research would benefit from greater attention to broader socioecological factors, particularly economic inequality, as potentially relevant contextual correlates of student outcomes.

Similar to national‐level perseverance results, economic inequality also significantly explains the variation in individual‐level academic perseverance, albeit with a smaller effect. Consistent with our a priori analysis estimates, economic inequality explains approximately 1% of the variance in academic perseverance. Although modest, this association may still be noteworthy because economic inequality represents a distal contextual factor operating broadly across national educational settings (Chua et al., 2019; King et al., 2022; Talhelm et al., 2014).

Furthermore, economic inequality was positively associated with academic perseverance across all four PISA waves spanning 22 years. In contrast, other macroeconomic indicators, including GDP per capita and unemployment, showed less consistent patterns of significance and direction across waves. This pattern suggests that the distribution of economic resources may be more consistently associated with students' academic perseverance than aggregate indicators of national economic conditions. Taken together, the findings support the cross‐wave consistency of the positive association.

A surprising finding is that economic inequality is positively associated with students' academic perseverance. Living in economically unequal contexts can feel terrible. If people fail in the more intense academic competition (Sommet et al., 2023) or experience stronger anxiety triggered by social conditions (King et al., 2024) of inequality, they might give up on persisting with their studies. Reduced intergenerational mobility resulting from inequality may discourage students and lower their expectations of education's future returns (Browman et al., 2017). Despite these potentially discouraging conditions, students in more unequal countries reported higher levels of academic perseverance. This pattern suggests that the relationship between economic inequality and students' educational responses may be more complex than a uniformly detrimental account would imply.

One possible theoretical interpretation is that the positive association between economic inequality and academic perseverance may reflect, at least in part, an adaptive response to unequal environments. Previous research has extensively documented the adverse consequences of inequality for educational outcomes (Chiu & Chow, 2015; Elgar et al., 2019; King et al., 2022, 2024), whereas comparatively less attention has been paid to how students may respond to and navigate such conditions. The present findings are consistent with Sommet's inequality–competition framework (Sommet et al., 2024), which proposes that economic inequality heightens perceived competition and may activate both approach‐ and avoidance‐oriented responses, thereby giving rise to potentially adaptive as well as maladaptive outcomes. Within this framework, academic perseverance may represent one possible behavioural manifestation of an approach‐oriented response to inequality.

Taken together, these findings further highlight the complexity of outcomes associated with economic inequality. Existing evidence suggests that economic inequality may be associated with seemingly contradictory outcomes: it may promote both competition and cooperation (Sommet et al., 2024); increase depression and anxiety while also being associated with pride and resilience (Pickett & Wilkinson, 2015; Sommet et al., 2024); and be linked to disengagement as well as persistence (Klimm, 2019). These divergent patterns suggest that the relationship between economic inequality and individual outcomes may be more complex than previously conceptualized, warranting further investigation into the mechanisms and conditions underlying these different responses, particularly in educational contexts.

This complexity may partly reflect differences in students' socioeconomic circumstances. In the present study, family SES moderated the association between economic inequality and academic perseverance in three of the four PISA waves, with the positive association being stronger among students from lower‐SES families. One possible explanation is that lower‐SES students are more likely to experience educational and material disadvantages, including less favourable learning environments, fewer educational resources and a weaker sense of school belonging (Hair et al., 2015; King et al., 2022; Rakesh et al., 2025). Repeated exposure to such challenges may be associated with the development of more persistence‐oriented ways of coping with academic demands. In addition, education may represent an especially important pathway for lower‐SES students to improve their socioeconomic circumstances, making academic perseverance particularly salient for them. This interpretation is broadly consistent with adult research suggesting that lower‐SES individuals may exhibit stronger aspirations for wealth, status and self‐improvement under conditions of greater inequality (Loughnan et al., 2011; Wang et al., 2023).

### Is greater perseverance in unequal contexts necessarily beneficial?

Although academic perseverance is generally associated with favourable educational outcomes (Credé et al., 2017; Lam & Zhou, 2022; Sudina & Plonsky, 2021), the positive association observed in the present study should not be interpreted as evidence that economic inequality is beneficial for students. Perseverance is only one component of educational success, and greater persistence does not necessarily translate into stronger academic achievement or improved opportunities, particularly when students face unequal access to educational resources and support.

This issue may be especially important for students from lower‐SES families. These students may invest substantial effort while continuing to experience structural disadvantages that constrain the returns to that effort (Chmielewski & Reardon, 2016). The finding that the association between inequality and perseverance was stronger among lower‐SES students therefore should not be taken to imply that inequality benefits them. Rather, it highlights the importance of ensuring that students' efforts are supported by adequate educational resources and institutional conditions. Policies that reduce disparities in school funding and material support may help ensure that perseverance is more likely to translate into meaningful educational opportunities (Jackson et al., 2016; Lafortune et al., 2018).

The findings also raise questions about the motivations and costs underlying perseverance in unequal contexts. Persistence may arise from different sources, including intrinsic interest, aspirations for upward mobility, perceived competition or external social and family expectations. These forms of perseverance may have different consequences for achievement and well‐being. For example, persistence accompanied by excessive pressure or anxiety may carry psychological costs even when it sustains academic engagement (King et al., 2024; Pickett & Wilkinson, 2015). Future research should therefore distinguish between different motivations for perseverance and examine when perseverance in unequal environments is associated with beneficial outcomes, when it entails substantial costs and whether students' efforts are ultimately rewarded.

### Limitations

This study has several limitations. First, given the observational nature of the study, the findings should not be interpreted causally. Second, we used only objective Gini coefficients to measure inequality; subjective perceptions of inequality may yield different results, as students might respond more to proximal competitive environments than to abstract inequality indicators. Third, we focused on national‐level inequality, whereas regional inequality patterns may have distinct structural characteristics and impacts. Fourth, our cross‐sectional design across four PISA waves prevents examination of within‐individual changes in perseverance as inequality fluctuates over time. Longitudinal studies tracking the same individuals would provide valuable insights into the dynamic relationship between inequality and perseverance. Moreover, because some countries participated in multiple PISA waves, the findings across the four waves should not be regarded as fully independent replications, and the recurrence of countries may partly contribute to the observed cross‐wave consistency. In addition, although the final student and school sampling weights were incorporated, PISA replicate weights could not be directly accommodated in the multilevel modelling framework used in this study. Consequently, the reported standard errors may not fully account for the complex sampling design. Additionally, because PISA samples only 15‐year‐olds who remain enrolled in school, differential dropout and school coverage across countries may introduce selection bias. In high‐inequality countries, low‐SES students who remain in school may constitute a positively selected subgroup with relatively higher perseverance, which could partly contribute to the observed moderation by family SES.

A further limitation concerns the cross‐wave comparability of the perseverance measures. Because PISA used different items and response scales across the four cycles, the measures may not be fully equivalent. Moreover, as perseverance was self‐reported, cross‐wave and cross‐national differences may partly reflect differences in item interpretation and response styles. Future research should replicate these findings using more consistent and objective measures of perseverance.

The level of inference also warrants consideration. Although the multilevel models examined the association between country‐level economic inequality and student‐level academic perseverance, the focal predictor varied only at the country level. The supplementary hybrid (Mundlak) analysis showed that both the between‐country and within‐country, across‐wave components of Gini were positively associated with academic perseverance (Table S7), suggesting that the association was not entirely attributable to stable between‐country differences. However, the within‐country component reflects country‐level variation across PISA waves rather than within‐person change, and different measures of academic perseverance were used across waves. Accordingly, this analysis should be interpreted as supplementary evidence, and caution is warranted when interpreting both its results and the proposed individual‐level psychological mechanisms.

Finally, self‐reported academic perseverance may conceptually overlap with broader cultural orientations, particularly Long‐Term Orientation, which partly incorporates perseverance‐ and persistence‐related content. Cross‐national differences may therefore reflect not only academic perseverance, but also cultural norms and systematic response styles. Although the main findings remained positive and were statistically significant in most cases after additionally adjusting for Long‐Term Orientation, Individualism, Uncertainty Avoidance, cultural tightness and country‐level extreme and acquiescent response styles (Tables S8–S11, S13–S14), these analyses were still limited by the number of countries with available data and potential collinearity among country‐level indicators, and they cannot fully rule out cultural confounding. Future research could incorporate a broader range of cross‐cultural indicators and alternative assessment methods to further examine the roles of cultural context and response styles in the association between economic inequality and academic perseverance.

## CONCLUSION

Drawing on 22 years of cross‐national data from 73 countries, this study shows that economic inequality is positively associated with students' self‐reported academic perseverance. In three of the four PISA waves, this association was stronger among students from lower‐SES backgrounds. These findings suggest that the educational correlates of inequality may be more complex than uniformly negative accounts imply, without indicating that inequality itself is beneficial. One possible interpretation is that perseverance may reflect an approach‐oriented response in unequal contexts. Future research should examine the motivations, costs and longer‐term consequences of such perseverance, while policy efforts should continue to reduce structural disadvantage and support students' educational opportunities.

## AUTHOR CONTRIBUTIONS

**Yifan Wang:** Conceptualization; investigation; writing – original draft; writing – review and editing; visualization; methodology; formal analysis; data curation. **Yanru Liu:** Visualization; writing – review and editing; writing – original draft; validation; methodology. **Dennis Alonzo:** Conceptualization; investigation; funding acquisition; writing – original draft; writing – review and editing; project administration; supervision; resources.

## CONFLICT OF INTEREST STATEMENT

None declared.

## ETHICS STATEMENT

This research has been approved by the Ethics Committee of the University of New South Wales (iRECS 8431).

## DECLARATION OF AI AND AI‐ASSISTED TECHNOLOGIES IN THE WRITING PROCESS

During the preparation of this work, the authors utilized Claude Sonnet 3.7 to refine and correct the language, ensure linguistic clarity and minimize grammatical mistakes. After this tool was used, the authors reviewed and edited the content as needed. The authors take full responsibility for the content of the publication.

## Supporting information


Data S1

## Data Availability

The preregistration report, dataset and analysis code can be found on the Open Science Framework (OSF) at https://osf.io/xfm9q/?view_only=21bd84bb5ce14733a6810f01c5937b0e.
