# Supplementary material for: Economic inequality and academic perseverance: Evidence from 73 countries
Source: Br J Educ Psychol. 2026 Sep 15;96(4):1862–83. doi: 10.1111/bjep.70124 (PMC13752663; doi:10.1111/bjep.70124)
Supplement: Supplementary file 1 [file BJEP-96-1862-s001.docx]

**Table S1.** Control Variables Table

| **Variable** | **Level** | **Coding/Measurement** | **Data Source** | **Data Year** |
| --- | --- | --- | --- | --- |
| Gender | Student | -0.5 = female; 0.5 = male | PISA Student Data | 2022, 2018, 2012, 2000 |
| Age | Student | Continuous variable | PISA Student Data |  |
| Origin | Student | -0.5 = none of the parents born in the country of the test; 0.5 = at least one parent is born in the country of the test | PISA Student Data |  |
| Total Population | National | Number of people | World Bank: Population, total | 2020, 2016, 2010, 1998 |
| Unemployment Rate | National | Percentage | World Bank: Unemployment, total |  |
| GDP per capita | National | USD per capita | World Bank: GDP per capita |  |

*Note.* National-level variables prioritize x-2 year data, with most recent available estimates from prior years used when current year data is unavailable. Data sources: Student-level data from PISA database, national-level data from World Bank (WB) database.

**Table S2**. Multiple Imputation Results for Missing Data Analysis

| **Variable** | **PISA 2022** | | | **PISA 2018** | | | **PISA 2012** | | | **PISA 2000** | | |
| --- | --- | --- | --- | --- | --- | --- | --- | --- | --- | --- | --- | --- |
|  | *B* | *SE* | *p* | *B* | *SE* | *p* | *B* | *SE* | *p* | *B* | *SE* | *p* |
| Intercept | 0.002 | 0.02 | 0.904 | 3.005 | 0.015 | < .001^***^ | 0.111 | 0.027 | < .001^***^ | 0.091 | 0.03 | .003^**^ |
| GINI Index | 0.016 | 0.004 | < .001^***^ | 0.01 | 0.002 | < .001^***^ | 0.016 | 0.005 | .001^**^ | 0.015 | 0.004 | < .001^***^ |
| SES | 0.108 | 0.002 | < .001^***^ | 0.05 | 0.001 | < .001^***^ | 0.108 | 0.002 | < .001^***^ | - | - | - |
| Log GDP | -0.061 | 0.06 | 0.306 | -0.164 | 0.039 | < .001^***^ | -0.416 | 0.074 | < .001^***^ | -0.098 | 0.088 | 0.265 |
| Gender | 0.092 | 0.003 | < .001^***^ | -0.087 | 0.002 | < .001^***^ | 0.026 | 0.004 | < .001^***^ | -0.184 | 0.009 | < .001^***^ |
| Age | 0.04 | 0.006 | < .001^***^ | -0.013 | 0.004 | .003^**^ | 0 | 0 | 0.303 | 0.001 | 0.001 | 0.339 |
| Immigration Origin | 0.073 | 0.006 | < .001*^**^ | 0.047 | 0.003 | < .001^***^ | -0.103 | 0.006 | < .001^***^ | 0.075 | 0.017 | < .001^***^ |
| Log Population | -0.019 | 0.031 | 0.551 | -0.021 | 0.023 | 0.357 | 0.336 | 0.075 | < .001^***^ | -0.113 | 0.056 | .044^*^ |
| Unemployment Rate | 0.002 | 0.006 | 0.677 | 0.003 | 0.003 | 0.405 | -0.005 | 0.006 | 0.386 | 0.033 | 0.01 | .001^**^ |
| GINI × SES | -0.003 | 0 | < .001^***^ | -0.002 | 0 | < .001^***^ | -0.002 | 0 | < .001^***^ | - | - | - |

Note. *B* = unstandardized regression coefficient; *SE* = standard error. All continuous variables were grand-mean centered prior to analysis. All models included random intercepts for country and school levels (Country/School) to account for the nested structure of the data. Parameter estimates were pooled across imputed datasets using Rubin's rules via the testEstimates() function. The data and measurements used are the same as those of the analysis in the main text.. ^*^*p* < .05. ^**^*p* < .01. ^***^*p* < .001.

**Table S3.** OLS Regression Results with Robust Standard Errors and Country Clustering

| Measure | Study A | | | | | Study B | | | | | Study C | | | | | Study D | | | | |
| --- | --- | --- | --- | --- | --- | --- | --- | --- | --- | --- | --- | --- | --- | --- | --- | --- | --- | --- | --- | --- |
|  | (PISA 2022) | | | | | (PISA 2018) | | | | | (PISA 2012) | | | | | (PISA 2000) | | | | |
| Description | 347150 / 13800 / 44 | | | | | 494559 / 20297 / 72 | | | | | 480174/ 17865 / 52 | | | | | 52510 / 2797 / 22 | | | | |
| Variable | *B* | *SE* | *t* | *p* | 95% CI | *B* | *SE* | *t* | *p* | 95% CI | *B* | *SE* | *t* | *p* | 95% CI | *B* | *SE* | *t* | *p* | 95% CI |
| Intercept | 0.01 | 0.02 | 0.51 | 0.611 | [-0.03, 0.05] | 3.04^***^ | 0.07 | 44.03 | < .001 | [2.90, 3.18] | 0.11^***^ | 0.02 | 5.24 | < .001 | [0.07, 0.16] | 0.09^**^ | 0.03 | 3.42 | 0.003 | [0.04, 0.15] |
| GINI | 0.01^**^ | 0.00 | 3.71 | 0.001 | [0.01, 0.02] | 0.01^***^ | 0 | 4.63 | < .001 | [0.01, 0.02] | 0.01^*^ | 0.00 | 2.63 | 0.011 | [0.00, 0.02] | - | - | - | - | - |
| ESCS | 0.10^***^ | 0.01 | 13.97 | < .001 | [0.08, 0.11] | 0.03^***^ | 0.01 | 5.41 | < .001 | [0.02, 0.04] | 0.11^***^ | 0.01 | 10.13 | < .001 | [0.09, 0.13] | 0.03^*^ | 0.01 | 2.77 | 0.011 | [0.01, 0.04] |
| Log GDP | -0.06 | 0.06 | -1.12 | 0.27 | [-0.18, 0.05] | -0.13^**^ | 0.04 | -3.61 | 0.001 | [-0.20, -0.06] | -0.40^***^ | 0.08 | -4.98 | < .001 | [-0.56, -0.24] | -0.1 | 0.1 | -0.98 | 0.337 | [-0.32, 0.11] |
| Gender | 0.09^***^ | 0.02 | 5.69 | < .001 | [0.06, 0.12] | -0.08^***^ | 0.01 | -7.49 | < .001 | [-0.10, -0.06] | 0.03 | 0.02 | 1.71 | 0.093 | [-0.01, 0.07] | -0.18^***^ | 0.02 | -9.65 | < .001 | [-0.22, -0.14] |
| Age | 0.06^***^ | 0.01 | 4.63 | < .001 | [0.04, 0.09] | -0.01 | 0.03 | -0.16 | 0.87 | [-0.07, 0.06] | 0.00^**^ | 0.00 | 3.25 | 0.002 | [0.00, 0.00] | 0.00 | 0.00 | 2.04 | 0.054 | [-0.00, 0.01] |
| Origin | 0.09^**^ | 0.02 | 3.94 | < .001 | [0.04, 0.14] | 0.03 | 0.02 | 1.82 | 0.076 | [-0.00, 0.07] | -0.14^***^ | 0.03 | -4.35 | < .001 | [-0.21, -0.08] | 0.09^*^ | 0.04 | 2.31 | 0.031 | [0.01, 0.17] |
| Log Population | -0.01 | 0.03 | -0.41 | 0.68 | [-0.08, 0.05] | -0.02 | 0.04 | -0.51 | 0.615 | [-0.09, 0.06] | 0.36^***^ | 0.08 | 4.56 | < .001 | [0.20, 0.52] | -0.13^*^ | 0.05 | -2.7 | 0.013 | [-0.24, -0.03] |
| Unemployment | 0.00 | 0.00 | 0.3 | 0.765 | [-0.01, 0.01] | 0.01^**^ | 0.00 | 3.41 | 0.001 | [0.00, 0.02] | 0.00 | 0.00 | 0.33 | 0.744 | [-0.01, 0.01] | 0.03^**^ | 0.01 | 3.3 | 0.003 | [0.01, 0.05] |
| GINI × ESCS | -0.001^**^ | 0.00 | -3.37 | 0.002 | [-0.01, -0.00] | 0.00 | 0.00 | -1.35 | 0.183 | [-0.00, 0.00] | 0.00 | 0.00 | -1.57 | 0.122 | [-0.00, 0.00] | - | - | - | - | - |

Note. B = unstandardized regression coefficient; SE = standard error; CI = confidence interval. Description row indicates total sample size / school-level observations / number of countries for each study. Robust standard errors clustered by country. All variables were mean-centered prior to analysis. Dashes (—) indicate variables not included in the respective model. GINI represents income inequality coefficient; ESCS = economic, social and cultural status; GDP = gross domestic product. Gender coded as male = 1, female = 0. Origin represents immigrant background of parents. The GINI × ESCS interaction term was not included in the PISA 2000 model due to different variable specifications.

**Table S4.** Country-Level Multiple Regression Analysis Predicting Perseverance from Gini Index and Control Variables

|  | PISA 2022 | | | | | PISA 2018 | | | | | PISA 2012 | | | | | PISA 2000 | | | | |
| --- | --- | --- | --- | --- | --- | --- | --- | --- | --- | --- | --- | --- | --- | --- | --- | --- | --- | --- | --- | --- |
| Variable | *B* | *SE* | *t* | *p* | 95% CI | *B* | *SE* | *t* | *p* | 95% CI | *B* | *SE* | *t* | *p* | 95% CI | *B* | *SE* | *t* | *p* | 95% CI |
| Constant | -0.47 | 0.37 | -1.27 | .213 | [-1.21, 0.28] | 3.25^***^ | 0.25 | 13.09 | < .001 | [2.76, 3.75] | -0.35 | 0.35 | -0.98 | .330 | [-1.06, 0.36] | 0.39 | 0.6 | 0.65 | .527 | [-0.88, 1.67] |
| GINI Index | 0.01^**^ | 0.00 | 3.69 | .001 | [0.01, 0.02] | 0.01^***^ | 0.00 | 4.25 | < .001 | [0.01, 0.01] | 0.01^*^ | 0.01 | 2.42 | .019 | [0.00, 0.02] | 0.02^**^ | 0.00 | 3.69 | .002 | [0.01, 0.02] |
| Log GDP | 0.04 | 0.06 | 0.68 | .503 | [-0.08, 0.16] | -0.11^**^ | 0.04 | -3.12 | .003 | [-0.18, -0.04] | -0.34^***^ | 0.08 | -4.2 | < .001 | [-0.50, -0.18] | -0.06 | 0.09 | -0.72 | .483 | [-0.25, 0.12] |
| Log Population | -0.03 | 0.03 | -0.88 | .387 | [-0.09, 0.04] | -0.02 | 0.02 | -0.99 | .324 | [-0.06, 0.02] | 0.26^**^ | 0.09 | 2.82 | .007 | [0.07, 0.44] | -0.11 | 0.06 | -1.98 | .064 | [-0.23, 0.01] |
| Unemployment Rate | 0.00 | 0.01 | 0.43 | .667 | [-0.01, 0.02] | 0.00 | 0.00 | 0.99 | .328 | [0.00, 0.01] | 0.00 | 0.01 | 0.17 | .864 | [-0.01, 0.02] | 0.03^**^ | 0.01 | 3.38 | .004 | [0.01, 0.05] |

Note. PISA 2000: N = 22 countries, R² = .664, F(4, 17) = 8.392, p < .001, adjusted R² = .585. PISA 2012: N = 52 countries, R² = .460, F(4, 47) = 9.994, p < .001, adjusted R² = .414. PISA 2018: N = 71 countries, R² = .381, F(4, 66) = 10.18, p < .001, adjusted R² = .344. PISA 2022: N = 44 countries, R² = .397, F(4, 39) = 6.430, p < .001, adjusted R² = .336.

**Table S5.** Original and Revised Perseverance Items Across PISA Waves

| PISA Wave | No. | Original Item | Revised Item Set | Note |
| --- | --- | --- | --- | --- |
| PISA 2000 | 1 | I will work as hard as possible. | I will work as hard as possible. |  |
| PISA 2000 | 2 | I will keep working even if the material is difficult. | I will keep working even if the material is difficult. |  |
| PISA 2000 | 3 | I will try to do my best to acquire the knowledge and skills taught. | I will try to do my best to acquire the knowledge and skills taught. |  |
| PISA 2000 | 4 | I will put forth my best effort. | I will put forth my best effort. |  |
| PISA 2012 | 1 | When confronted with a problem, I give up easily. (R) | When confronted with a problem, I give up easily. (R) |  |
| PISA 2012 | 2 | I put off difficult problems. (R) | I put off difficult problems. (R) |  |
| PISA 2012 | 3 | I continue working on tasks until everything is perfect. | I continue working on tasks until everything is perfect. |  |
| PISA 2012 | 4 | When confronted with a problem, I do more than what is expected of me. | When confronted with a problem, I do more than what is expected of me. |  |
| PISA 2012 | 5 | I remain interested in the tasks that I start. | REMOVED | Reflects intrinsic interest; removed as passion item |
| PISA 2018 | 1 | I find satisfaction in working as hard as I can. | REMOVED | Reflects intrinsic satisfaction; removed as passion item |
| PISA 2018 | 2 | Once I start a task, I persist until it is finished. | Once I start a task, I persist until it is finished. |  |
| PISA 2018 | 3 | Part of the enjoyment I get from doing things is when I improve on my past performance. | REMOVED | Reflects enjoyment/intrinsic motivation; removed as passion item |
| PISA 2018 | 4 | If I am not good at something, I would rather keep struggling to master it than move on to something I may be good at. | If I am not good at something, I would rather keep struggling to master it than move on to something I may be good at. |  |
| PISA 2022 | 1 | I keep working on a task until it is finished. | I keep working on a task until it is finished. |  |
| PISA 2022 | 2 | I apply additional effort when work becomes challenging. | I apply additional effort when work becomes challenging. |  |
| PISA 2022 | 3 | I finish tasks that I started even when they become boring. | I finish tasks that I started even when they become boring. |  |
| PISA 2022 | 4 | I stop when work becomes too difficult. (R) | I stop when work becomes too difficult. (R) |  |
| PISA 2022 | 5 | I am more persistent than most people I know. | I am more persistent than most people I know. |  |
| PISA 2022 | 6 | I give up after making mistakes. (R) | I give up after making mistakes. (R) |  |
| PISA 2022 | 7 | I quit doing homework if it is too long. (R) | I quit doing homework if it is too long. (R) |  |
| PISA 2022 | 8 | I complete tasks even when they become more difficult than I thought. | I complete tasks even when they become more difficult than I thought. |  |
| PISA 2022 | 9 | I finish what I start. | I finish what I start. |  |
| PISA 2022 | 10 | I give up easily. (R) | I give up easily. (R) |  |

*Note.* (R) = reverse-scored item. Items in the revised set were screened based on their conceptual alignment with the perseverance component of grit. Items reflecting passion-related content, including intrinsic interest, enjoyment, or satisfaction, were removed (i.e., items containing keywords such as interested, enjoyment, and satisfaction). The remaining items capture persistence and effort, consistent with the conceptual definition of perseverance. Internal consistency of the revised scales was acceptable (PISA 2012: Cronbach's α = .76; PISA 2018: Cronbach's α = .64). For reference, the original scales reported the following internal consistency: PISA 2022: α = .77; PISA 2012: α = .78; PISA 2018: α = .93; PISA 2000: α = .77.

**Table S6.** Reanalysis Results: Economic Inequality and Academic Perseverance (Passion Items Excluded)

|  | Study B (PISA 2018) | | Study C (PISA 2012) | |
| --- | --- | --- | --- | --- |
|  | *B* | 95% CI | *B* | 95% CI |
| Gini Coefficient | 0.010^***^ | [0.006, 0.015] | 0.009^*^ | [0.001, 0.017] |
| SES | 0.027^***^ | [0.025, 0.030] | 0.087^***^ | [0.083, 0.090] |
| Age | 0.024^***^ | [0.018, 0.031] | 0.042^***^ | [0.032, 0.052] |
| Gender (+0.5 = Boys) | −0.031^***^ | [−0.035, −0.028] | 0.018^***^ | [0.012, 0.024] |
| Origin (+0.5 = Immigrant) | −0.047^***^ | [−0.060, −0.035] | −0.079^***^ | [−0.098, −0.060] |
| GDP (log) | −0.202^***^ | [−0.265, −0.138] | −0.280^***^ | [−0.416, −0.145] |
| National Population (log) | −0.031 | [−0.071, 0.009] | 0.239^***^ | [0.090, 0.388] |
| Unemployment Rate | 0.002 | [−0.003, 0.007] | −0.003 | [−0.015, 0.010] |
| Gini × SES | −0.001^***^ | [−0.001, −0.001] | −0.003^***^ | [−0.003, −0.002] |
| Intercept | 2.924^***^ | [2.901, 2.946] | 3.869^***^ | [3.817, 3.921] |
| N (students / countries) | 555,308 / 72 | | 480,174 / 52 | |

Note. PISA 2018: passion items ST182Q03HA and ST182Q05HA excluded; 2 items retained (ST182Q04HA, ST182Q06HA). PISA 2012: passion item ST93Q07 excluded; 4 items retained. *GDP* = gross domestic product per capita. *SES* = Socioeconomic Status. *CI* = Confidence Interval. * *p* < .05. ** *p* < .01. *** *p* < .001.

**Figure S1**

SES moderates the relationship between economic inequality and academic perseverance (without Passion)

\

*Note.* Lines represent predicted values at different socioeconomic status (SES) levels: +1 *SD* (solid), mean (dashed), and -1 *SD* (dotted). Shaded areas indicate 95% confidence interval.

**Figure S2**

Kernel Density Plots of Perseverance Score Distributions Across Four PISA Waves

*Note.* Kernel density plots comparing the distribution of perseverance scores between the analytic sample and the full PISA sample for each wave. Orange shading represents the analytic sample (after exclusions due to missing data); blue shading represents the full PISA sample. Dashed vertical lines indicate the respective means. Studies A, C, and D use the Weighted Likelihood Estimate (WLE) index expressed in logit units, which is calibrated against the OECD reference sample such that means naturally tend toward zero; Study B uses the raw mean score of four Likert-scale items (1–4), with the mean falling near the scale midpoin

**Table S7.** Hybrid (Mundlak) Multilevel Estimates of the Between-Country and Within-Country Associations Between Economic Inequality and Academic Perseverance.

| Predictor | *b* | *SE* | 95% CI Lower | 95% CI Upper | *p* |
| --- | --- | --- | --- | --- | --- |
| Gini, between-country | 0.018 | 0.003 | 0.011 | 0.024 | < .001 |
| Gini, within-country | 0.015 | 0.001 | 0.013 | 0.017 | < .001 |
| PISA 2012 | -0.109 | 0.016 | -0.139 | -0.078 | < .001 |
| PISA 2018 | -0.043 | 0.008 | -0.058 | -0.028 | < .001 |
| PISA 2022 | -0.021 | 0.008 | -0.037 | -0.005 | .012 |
| ESCS | 0.076 | 0.001 | 0.074 | 0.078 | < .001 |
| Gender | -0.065 | 0.002 | -0.069 | -0.061 | < .001 |
| Age | 0.049 | 0.003 | 0.043 | 0.055 | < .001 |
| GDP, within-country | 0.000 | 0.008 | -0.015 | 0.015 | .974 |
| GDP, between-country | -0.065 | 0.037 | -0.138 | 0.007 | .083 |
| Population, within-country | -0.053 | 0.031 | -0.115 | 0.009 | .091 |
| Population, between-country | -0.018 | 0.032 | -0.082 | 0.045 | .574 |
| Unemployment, within-country | 0.007 | 0.001 | 0.005 | 0.009 | < .001 |
| Unemployment, between-country | 0.004 | 0.005 | -0.006 | 0.013 | .459 |

*Note.* The hybrid (Mundlak) multilevel model simultaneously included country-mean Gini (between-country component) and country-mean-centered Gini (within-country, across-wave component). Academic perseverance scores were standardized separately within each PISA wave prior to pooling because the perseverance measures differed across cycles. The model was estimated at the student level using student sampling weights and included PISA wave indicators, ESCS, gender, age, and within- and between-country components of GDP, population size, and unemployment as covariates. Students were nested within schools and countries. *N* = 1,121,211 students from 73 countries. PISA 2000 served as the reference category.

**Table S8.** Multilevel Sensitivity Analyses of the Association Between Economic Inequality and Academic Perseverance After Adjusting for Long-Term Orientation Across Four PISA Waves

| Study | Variable | *b* | *SE* | CI_lower | CI_upper | *p* |
| --- | --- | --- | --- | --- | --- | --- |
| PISA 2022 | GINI2020 | 0.006 | 0.005 | -0.003 | 0.015 | .220 |
|  | ESCS_cwc | 0.106 | 0.008 | 0.091 | 0.122 | < .001 |
|  | ESCS_cm | -0.241 | 0.096 | -0.429 | -0.054 | .017 |
|  | logGDP2020 | 0.192 | 0.093 | 0.009 | 0.375 | .048 |
|  | logPopulation2020 | -0.037 | 0.036 | -0.108 | 0.033 | .305 |
|  | Unemployment2020 | 0.002 | 0.006 | -0.011 | 0.014 | .795 |
|  | Gender | 0.091 | 0.004 | 0.084 | 0.098 | < .001 |
|  | Age | 0.041 | 0.007 | 0.029 | 0.054 | < .001 |
|  | Origin | 0.061 | 0.008 | 0.046 | 0.076 | < .001 |
|  | LTO | -0.001 | 0.001 | -0.003 | 0.001 | .426 |
|  | GINI2020:ESCS_cwc | -0.003 | 0.001 | -0.005 | -0.001 | .002 |
| PISA 2018 | GINI2016 | 0.005 | 0.003 | -0.001 | 0.012 | .099 |
|  | ESCS_cwc | 0.050 | 0.004 | 0.042 | 0.057 | < .001 |
|  | ESCS_cm | -0.011 | 0.053 | -0.115 | 0.093 | .843 |
|  | logGDP2016 | -0.128 | 0.056 | -0.237 | -0.018 | .026 |
|  | logPopulation2016 | -0.008 | 0.025 | -0.058 | 0.041 | .751 |
|  | Unemployment2016 | 0.005 | 0.003 | 0.000 | 0.011 | .077 |
|  | Gender | -0.075 | 0.002 | -0.079 | -0.072 | < .001 |
|  | Age | -0.033 | 0.003 | -0.040 | -0.027 | < .001 |
|  | Origin | 0.049 | 0.004 | 0.040 | 0.057 | < .001 |
|  | LTO | -0.002 | 0.001 | -0.003 | 0.000 | .057 |
|  | GINI2016:ESCS_cwc | -0.001 | 0.001 | -0.002 | 0.000 | .048 |
| PISA 2012 | GINI2010 | 0.014 | 0.006 | 0.003 | 0.026 | .022 |
|  | ESCS_cwc | 0.106 | 0.009 | 0.089 | 0.123 | < .001 |
|  | ESCS_cm | 0.136 | 0.117 | -0.093 | 0.366 | .251 |
|  | logGDP2010 | -0.427 | 0.138 | -0.699 | -0.156 | .004 |
|  | logPopulation2010 | 0.381 | 0.157 | 0.074 | 0.688 | .019 |
|  | Unemployment2010 | -0.001 | 0.007 | -0.015 | 0.013 | .877 |
|  | Gender | 0.027 | 0.004 | 0.020 | 0.035 | <.001 |
|  | Age | 0.060 | 0.007 | 0.047 | 0.074 | <.001 |
|  | Origin | -0.127 | 0.013 | -0.151 | -0.102 | <.001 |
|  | LTO | -0.002 | 0.001 | -0.005 | 0.001 | .122 |
|  | GINI2010:ESCS_cwc | -0.003 | 0.001 | -0.005 | 0.000 | .037 |
| PISA 2000 | GINI1998 | 0.023 | 0.005 | 0.014 | 0.032 | <.001 |
|  | ESCS_cwc | 0.094 | 0.018 | 0.060 | 0.129 | <.001 |
|  | ESCS_cm | 0.199 | 0.126 | -0.048 | 0.447 | .138 |
|  | logGDP1998 | -0.269 | 0.148 | -0.559 | 0.021 | .088 |
|  | logPopulation1998 | -0.181 | 0.057 | -0.293 | -0.068 | .007 |
|  | Unemployment1998 | 0.024 | 0.010 | 0.005 | 0.043 | .028 |
|  | Gender | -0.218 | 0.009 | -0.235 | -0.200 | <.001 |
|  | Age | 0.007 | 0.001 | 0.004 | 0.010 | <.001 |
|  | Origin | 0.110 | 0.021 | 0.069 | 0.151 | <.001 |
|  | LTO | 0.003 | 0.002 | 0.000 | 0.006 | .079 |
|  | GINI1998:ESCS_cwc | -0.002 | 0.002 | -0.005 | 0.001 | .243 |

*Note.* Unstandardized regression coefficients are reported. CI = confidence interval; ESCS = index of economic, social, and cultural status; ESCS_cwc = country-mean-centered student-level ESCS; ESCS_cm = country mean ESCS; LTO = Long-Term Orientation. Gini coefficients and continuous covariates were grand-mean centered, whereas ESCS was centered within countries. All models included student sampling weights, random intercepts for schools and countries, and a random slope for ESCS_cwc across countries. The Gini × ESCS_cwc term represents the cross-level interaction between country-level economic inequality and individual-level family socioeconomic status. LTO was included as a country-level cultural covariate. Models were estimated separately for PISA 2000, 2012, 2018, and 2022.

**Table S9.** Multilevel Sensitivity Analyses of the Association Between Economic Inequality and Academic Perseverance After Adjusting for Individualism Across Four PISA Waves

| Study | Variable | *b* | *SE* | CI_lower | CI_upper | *p* |
| --- | --- | --- | --- | --- | --- | --- |
| PISA 2022 | GINI2020 | 0.010 | 0.004 | 0.001 | 0.018 | .036 |
|  | ESCS_cwc | 0.105 | 0.008 | 0.089 | 0.121 | <.001 |
|  | ESCS_cm | -0.143 | 0.094 | -0.327 | 0.041 | .140 |
|  | logGDP2020 | 0.319 | 0.105 | 0.113 | 0.524 | .005 |
|  | logPopulation2020 | 0.009 | 0.039 | -0.069 | 0.086 | .830 |
|  | Unemployment2020 | 0.000 | 0.006 | -0.012 | 0.011 | .976 |
|  | Gender | 0.092 | 0.004 | 0.085 | 0.100 | <.001 |
|  | Age | 0.042 | 0.007 | 0.028 | 0.055 | <.001 |
|  | Origin | 0.062 | 0.008 | 0.046 | 0.077 | <.001 |
|  | IDV | -0.004 | 0.001 | -0.006 | -0.001 | .013 |
|  | GINI2020:ESCS_cwc | -0.003 | 0.001 | -0.006 | -0.001 | .004 |
| PISA 2018 | GINI2016 | 0.008 | 0.003 | 0.002 | 0.014 | .013 |
|  | ESCS_cwc | 0.047 | 0.004 | 0.038 | 0.055 | <.001 |
|  | ESCS_cm | -0.019 | 0.056 | -0.128 | 0.090 | .732 |
|  | logGDP2016 | 0.003 | 0.076 | -0.146 | 0.153 | .966 |
|  | logPopulation2016 | 0.000 | 0.026 | -0.051 | 0.051 | .995 |
|  | Unemployment2016 | 0.004 | 0.004 | -0.003 | 0.012 | .287 |
|  | Gender | -0.075 | 0.002 | -0.078 | -0.071 | <.001 |
|  | Age | -0.033 | 0.004 | -0.040 | -0.026 | <.001 |
|  | Origin | 0.049 | 0.004 | 0.041 | 0.058 | <.001 |
|  | IDV | -0.003 | 0.001 | -0.005 | -0.001 | .008 |
|  | GINI2016:ESCS_cwc | -0.001 | 0.001 | -0.002 | 0.000 | .046 |
| PISA 2012 | GINI2010 | 0.019 | 0.005 | 0.009 | 0.030 | .001 |
|  | ESCS_cwc | 0.105 | 0.009 | 0.088 | 0.122 | <.001 |
|  | ESCS_cm | 0.111 | 0.131 | -0.146 | 0.367 | .403 |
|  | logGDP2010 | -0.460 | 0.163 | -0.779 | -0.140 | .007 |
|  | logPopulation2010 | 0.364 | 0.173 | 0.025 | 0.704 | .041 |
|  | Unemployment2010 | -0.006 | 0.008 | -0.021 | 0.009 | .468 |
|  | Gender | 0.028 | 0.004 | 0.020 | 0.036 | <.001 |
|  | Age | 0.060 | 0.007 | 0.046 | 0.073 | <.001 |
|  | Origin | -0.126 | 0.013 | -0.151 | -0.101 | <.001 |
|  | IDV | 0.001 | 0.002 | -0.002 | 0.005 | .477 |
|  | GINI2010:ESCS_cwc | -0.003 | 0.001 | -0.005 | 0.000 | .028 |
| PISA 2000 | GINI1998 | 0.020 | 0.004 | 0.011 | 0.029 | .001 |
|  | ESCS_cwc | 0.092 | 0.018 | 0.057 | 0.127 | <.001 |
|  | ESCS_cm | 0.257 | 0.138 | -0.014 | 0.528 | .087 |
|  | logGDP1998 | 0.030 | 0.201 | -0.365 | 0.425 | .885 |
|  | logPopulation1998 | -0.037 | 0.074 | -0.182 | 0.108 | .625 |
|  | Unemployment1998 | 0.020 | 0.009 | 0.002 | 0.039 | .053 |
|  | Gender | -0.217 | 0.009 | -0.235 | -0.199 | <.001 |
|  | Age | 0.007 | 0.001 | 0.005 | 0.010 | <.001 |
|  | Origin | 0.110 | 0.021 | 0.068 | 0.152 | <.001 |
|  | IDV | -0.004 | 0.003 | -0.011 | 0.003 | .241 |
|  | GINI1998:ESCS_cwc | -0.002 | 0.002 | -0.005 | 0.001 | .275 |

*Note.* Unstandardized regression coefficients are reported. CI = confidence interval; ESCS = index of economic, social, and cultural status; ESCS_cwc = student-level ESCS centered within countries; ESCS_cm = country-mean ESCS; IDV = individualism. The Gini coefficient and continuous covariates were grand-mean centered, whereas ESCS was group-mean centered within countries. All models incorporated student sampling weights and included random intercepts for schools and countries, as well as a random slope for ESCS_cwc across countries. The Gini × ESCS_cwc term represents the cross-level interaction between country-level economic inequality and individual-level family socioeconomic status. IDV was included as a country-level cultural covariate. Models were estimated separately using PISA 2000, 2012, 2018, and 2022 data.

**Table S10.** Multilevel Sensitivity Analyses of the Association Between Economic Inequality and Academic Perseverance After Adjusting for Uncertainty Avoidance Across Four PISA Waves

| Study | Variable | *b* | *SE* | CI_lower | CI_upper | *p* |
| --- | --- | --- | --- | --- | --- | --- |
| PISA 2022 | GINI2020 | 0.011 | 0.005 | 0.001 | 0.020 | .031 |
|  | ESCS_cwc | 0.105 | 0.008 | 0.089 | 0.121 | <.001 |
|  | ESCS_cm | -0.229 | 0.098 | -0.420 | -0.037 | .026 |
|  | logGDP2020 | 0.265 | 0.112 | 0.046 | 0.485 | .024 |
|  | logPopulation2020 | -0.036 | 0.039 | -0.112 | 0.040 | .359 |
|  | Unemployment2020 | 0.000 | 0.006 | -0.012 | 0.013 | .950 |
|  | Gender | 0.092 | 0.004 | 0.085 | 0.100 | <.001 |
|  | Age | 0.042 | 0.007 | 0.029 | 0.055 | <.001 |
|  | Origin | 0.062 | 0.008 | 0.046 | 0.077 | <.001 |
|  | UAI | 0.001 | 0.001 | -0.001 | 0.003 | .331 |
|  | GINI2020:ESCS_cwc | -0.003 | 0.001 | -0.006 | -0.001 | .004 |
| PISA 2018 | GINI2016 | 0.009 | 0.003 | 0.003 | 0.015 | .009 |
|  | ESCS_cwc | 0.047 | 0.004 | 0.038 | 0.055 | <.001 |
|  | ESCS_cm | -0.059 | 0.059 | -0.175 | 0.056 | .321 |
|  | logGDP2016 | -0.058 | 0.076 | -0.208 | 0.092 | .452 |
|  | logPopulation2016 | -0.015 | 0.027 | -0.068 | 0.038 | .577 |
|  | Unemployment2016 | 0.002 | 0.004 | -0.006 | 0.011 | .587 |
|  | Gender | -0.075 | 0.002 | -0.078 | -0.071 | <.001 |
|  | Age | -0.033 | 0.004 | -0.040 | -0.026 | <.001 |
|  | Origin | 0.049 | 0.004 | 0.041 | 0.058 | <.001 |
|  | UAI | 0.001 | 0.001 | -0.001 | 0.003 | .241 |
|  | GINI2016:ESCS_cwc | -0.001 | 0.001 | -0.002 | 0.000 | .046 |
| PISA 2012 | GINI2010 | 0.020 | 0.005 | 0.010 | 0.031 | <.001 |
|  | ESCS_cwc | 0.105 | 0.009 | 0.088 | 0.122 | <.001 |
|  | ESCS_cm | 0.146 | 0.125 | -0.100 | 0.391 | .251 |
|  | logGDP2010 | -0.438 | 0.155 | -0.741 | -0.134 | .007 |
|  | logPopulation2010 | 0.350 | 0.169 | 0.020 | 0.681 | .044 |
|  | Unemployment2010 | -0.003 | 0.007 | -0.017 | 0.012 | .704 |
|  | Gender | 0.028 | 0.004 | 0.020 | 0.036 | <.001 |
|  | Age | 0.060 | 0.007 | 0.046 | 0.073 | <.001 |
|  | Origin | -0.126 | 0.013 | -0.151 | -0.101 | <.001 |
|  | UAI | -0.002 | 0.001 | -0.004 | 0.001 | .189 |
|  | GINI2010:ESCS_cwc | -0.003 | 0.001 | -0.005 | 0.000 | .029 |
| PISA 2000 | GINI1998 | 0.022 | 0.005 | 0.013 | 0.031 | <.001 |
|  | ESCS_cwc | 0.092 | 0.018 | 0.057 | 0.127 | <.001 |
|  | ESCS_cm | 0.194 | 0.134 | -0.068 | 0.457 | .170 |
|  | logGDP1998 | -0.092 | 0.187 | -0.459 | 0.275 | .630 |
|  | logPopulation1998 | -0.088 | 0.060 | -0.205 | 0.030 | .166 |
|  | Unemployment1998 | 0.020 | 0.011 | -0.001 | 0.040 | .084 |
|  | Gender | -0.217 | 0.009 | -0.235 | -0.199 | <.001 |
|  | Age | 0.007 | 0.001 | 0.005 | 0.010 | <.001 |
|  | Origin | 0.110 | 0.021 | 0.068 | 0.152 | <.001 |
|  | UAI | 0.000 | 0.002 | -0.004 | 0.003 | .894 |
|  | GINI1998:ESCS_cwc | -0.002 | 0.002 | -0.005 | 0.001 | .278 |

*Note.* Unstandardized regression coefficients are reported. CI = confidence interval; ESCS = index of economic, social, and cultural status; ESCS_cwc = student-level ESCS centered within countries; ESCS_cm = country-mean ESCS; UAI = uncertainty avoidance. The Gini coefficient and continuous covariates were grand-mean centered, whereas ESCS was group-mean centered within countries. All models incorporated student sampling weights and included random intercepts for schools and countries, as well as a random slope for ESCS_cwc across countries. The Gini × ESCS_cwc term represents the cross-level interaction between country-level economic inequality and individual-level family socioeconomic status. UAI was included as a country-level cultural covariate. Models were estimated separately using PISA 2000, 2012, 2018, and 2022 data.

**Table S11.** Multilevel Sensitivity Analyses of the Association Between Economic Inequality and Academic Perseverance After Adjusting for Cultural Tightness Across Four PISA Waves

| Study | Variable | *b* | *SE* | CI_lower | CI_upper | *p* |
| --- | --- | --- | --- | --- | --- | --- |
| PISA 2022 | GINI2020 | 0.010 | 0.006 | -0.001 | 0.022 | .092 |
|  | ESCS_cwc | 0.107 | 0.009 | 0.089 | 0.125 | <.001 |
|  | ESCS_cm | -0.212 | 0.141 | -0.488 | 0.064 | .149 |
|  | logGDP2020 | 0.165 | 0.155 | -0.140 | 0.469 | .301 |
|  | logPopulation2020 | -0.075 | 0.052 | -0.176 | 0.027 | .162 |
|  | Unemployment2020 | 0.002 | 0.008 | -0.014 | 0.017 | .838 |
|  | Gender | 0.088 | 0.004 | 0.080 | 0.097 | <.001 |
|  | Age | 0.041 | 0.008 | 0.026 | 0.056 | <.001 |
|  | Origin | 0.063 | 0.009 | 0.045 | 0.081 | <.001 |
|  | Tightness | 0.176 | 0.173 | -0.164 | 0.515 | .323 |
|  | GINI2020:ESCS_cwc | -0.004 | 0.001 | -0.006 | -0.001 | .003 |
| PISA 2018 | GINI2016 | 0.014 | 0.004 | 0.006 | 0.023 | .002 |
|  | ESCS_cwc | 0.047 | 0.004 | 0.038 | 0.056 | <.001 |
|  | ESCS_cm | 0.044 | 0.082 | -0.116 | 0.204 | .595 |
|  | logGDP2016 | -0.123 | 0.087 | -0.295 | 0.048 | .168 |
|  | logPopulation2016 | -0.007 | 0.035 | -0.076 | 0.062 | .846 |
|  | Unemployment2016 | 0.003 | 0.004 | -0.005 | 0.010 | .453 |
|  | Gender | -0.070 | 0.002 | -0.074 | -0.066 | <.001 |
|  | Age | -0.033 | 0.004 | -0.040 | -0.026 | <.001 |
|  | Origin | 0.049 | 0.005 | 0.040 | 0.058 | <.001 |
|  | Tightness | 0.163 | 0.101 | -0.034 | 0.361 | .115 |
|  | GINI2016:ESCS_cwc | -0.001 | 0.001 | -0.003 | 0.000 | .052 |
| PISA 2012 | GINI2010 | 0.013 | 0.007 | -0.002 | 0.027 | .093 |
|  | ESCS_cwc | 0.111 | 0.010 | 0.091 | 0.131 | <.001 |
|  | ESCS_cm | 0.086 | 0.184 | -0.273 | 0.446 | .642 |
|  | logGDP2010 | -0.390 | 0.242 | -0.864 | 0.084 | .118 |
|  | logPopulation2010 | 0.353 | 0.289 | -0.214 | 0.920 | .233 |
|  | Unemployment2010 | -0.004 | 0.010 | -0.023 | 0.014 | .659 |
|  | Gender | 0.016 | 0.004 | 0.008 | 0.025 | <.001 |
|  | Age | 0.064 | 0.008 | 0.050 | 0.079 | <.001 |
|  | Origin | -0.104 | 0.014 | -0.132 | -0.077 | <.001 |
|  | Tightness | 0.092 | 0.202 | -0.304 | 0.489 | .652 |
|  | GINI2010:ESCS_cwc | -0.003 | 0.001 | -0.006 | -0.001 | .020 |
| PISA 2000 | GINI1998 | 0.027 | 0.006 | 0.015 | 0.039 | .002 |
|  | ESCS_cwc | 0.094 | 0.021 | 0.052 | 0.136 | <.001 |
|  | ESCS_cm | 0.307 | 0.189 | -0.063 | 0.677 | .141 |
|  | logGDP1998 | -0.201 | 0.277 | -0.744 | 0.341 | .487 |
|  | logPopulation1998 | -0.100 | 0.074 | -0.246 | 0.045 | .212 |
|  | Unemployment1998 | 0.020 | 0.013 | -0.005 | 0.045 | .149 |
|  | Gender | -0.220 | 0.011 | -0.241 | -0.199 | <.001 |
|  | Age | 0.007 | 0.002 | 0.004 | 0.010 | <.001 |
|  | Origin | 0.112 | 0.025 | 0.064 | 0.160 | <.001 |
|  | Tightness | 0.262 | 0.223 | -0.175 | 0.699 | .271 |
|  | GINI1998:ESCS_cwc | -0.002 | 0.002 | -0.005 | 0.002 | .352 |

*Note.* Unstandardized regression coefficients are reported. CI = confidence interval; ESCS = index of economic, social, and cultural status; ESCS_cwc = student-level ESCS centered within countries; ESCS_cm = country-mean ESCS; Tightness = cultural tightness. The Gini coefficient and continuous covariates were grand-mean centered, whereas ESCS was group-mean centered within countries. All models incorporated student sampling weights and included random intercepts for schools and countries, as well as a random slope for ESCS_cwc across countries. The Gini × ESCS_cwc term represents the cross-level interaction between country-level economic inequality and individual-level family socioeconomic status. Cultural tightness was included as a country-level cultural covariate. Models were estimated separately using PISA 2000, 2012, 2018, and 2022 data.

**Table S12.** Items Used to Construct the Extreme Response Style and Acquiescent Response Style Indices Across Four PISA Waves

| PISA wave | Item code | Item content | Response categories | ERS coding | ARS coding |
| --- | --- | --- | --- | --- | --- |
| 2022 | ST034Q01TA | Agree/disagree: I feel like an outsider (or left out of things) at school. | 1-4 | Endpoint responses | Agreement: 1,2 |
|  | ST034Q02TA | Agree/disagree: I make friends easily at school. |  |  | Agreement: 1,2 |
|  | ST034Q03TA | Agree/disagree: I feel like I belong at school. |  |  | Agreement: 1,2 |
|  | ST034Q04TA | Agree/disagree: I feel awkward and out of place in my school. |  |  | Agreement: 1,2 |
|  | ST034Q05TA | Agree/disagree: Other students seem to like me. |  |  | Agreement: 1,2 |
|  | ST034Q06TA | Agree/disagree: I feel lonely at school. |  |  | Agreement: 1,2 |
|  | ST265Q01JA | Agree/disagree: I feel safe on my way to school. |  |  | Agreement: 1,2 |
|  | ST265Q02JA | Agree/disagree: I feel safe on my way home from school. |  |  | Agreement: 1,2 |
|  | ST265Q03JA | Agree/disagree: I feel safe in my classrooms at school. |  |  | Agreement: 1,2 |
|  | ST265Q04JA | Agree/disagree: I feel safe at other places at school (e.g. hallway, cafeteria, restroom). |  |  | Agreement: 1,2 |
|  | ST267Q01JA | Agree/disagree: The teachers at my school are respectful towards me. |  |  | Agreement: 3,4 |
|  | ST267Q02JA | Agree/disagree: If I walked into my classes upset, my teachers would be concerned about me. |  |  | Agreement: 3,4 |
|  | ST267Q03JA | Agree/disagree: If I came back to visit my school 3 years from now, my teachers would be excited to see me. |  |  | Agreement: 3,4 |
|  | ST267Q04JA | Agree/disagree: I feel intimidated by the teachers at my school. |  |  | Agreement: 3,4 |
|  | ST267Q05JA | Agree/disagree: When my teachers ask how I am doing, they are really interested in my answer. |  |  | Agreement: 3,4 |
|  | ST267Q06JA | Agree/disagree: The teachers at my school are friendly towards me. |  |  | Agreement: 3,4 |
|  | ST267Q07JA | Agree/disagree: The teachers at my school are interested in students' well-being. |  |  | Agreement: 3,4 |
|  | ST267Q08JA | Agree/disagree: The teachers at my school are mean towards me. |  |  | Agreement: 3,4 |
|  | ST268Q01JA | Agree/disagree: Mathematics is one of my favourite subjects. |  |  | Agreement: 3,4 |
|  | ST268Q02JA | Agree/disagree: [Test language] is one of my favourite subjects. |  |  | Agreement: 3,4 |
|  | ST268Q03JA | Agree/disagree: [Science] is one of my favourite subjects. |  |  | Agreement: 3,4 |
|  | ST268Q04JA | Agree/disagree: Mathematics is easy for me. |  |  | Agreement: 3,4 |
|  | ST268Q05JA | Agree/disagree: [Test language] is easy for me. |  |  | Agreement: 3,4 |
|  | ST268Q06JA | Agree/disagree: [Science] is easy for me. |  |  | Agreement: 3,4 |
|  | ST268Q07JA | Agree/disagree: I want to do well in my mathematics class. |  |  | Agreement: 3,4 |
|  | ST268Q08JA | Agree/disagree: I want to do well in my [test language] class. |  |  | Agreement: 3,4 |
|  | ST268Q09JA | Agree/disagree: I want to do well in my [science] class. |  |  | Agreement: 3,4 |
|  | ST292Q01JA | Agree/disagree: I often worry that it will be difficult for me in mathematics classes. |  |  | Agreement: 1,2 |
|  | ST292Q02JA | Agree/disagree: I get very tense when I have to do mathematics homework. |  |  | Agreement: 1,2 |
|  | ST292Q03JA | Agree/disagree: I get very nervous doing mathematics problems. |  |  | Agreement: 1,2 |
|  | ST292Q04JA | Agree/disagree: I feel helpless when doing a mathematics problem. |  |  | Agreement: 1,2 |
|  | ST292Q05JA | Agree/disagree: I worry that I will get poor [marks] in mathematics. |  |  | Agreement: 1,2 |
|  | ST292Q06JA | Agree/disagree: I feel anxious about failing in mathematics. |  |  | Agreement: 1,2 |
| 2018 | ST034Q01TA | Thinking about your school: I feel like an outsider (or left out of things) at school. |  |  | Agreement: 1,2 |
|  | ST034Q02TA | Thinking about your school: I make friends easily at school. |  |  | Agreement: 1,2 |
|  | ST034Q03TA | Thinking about your school: I feel like I belong at school. |  |  | Agreement: 1,2 |
|  | ST034Q04TA | Thinking about your school: I feel awkward and out of place in my school. |  |  | Agreement: 1,2 |
|  | ST034Q05TA | Thinking about your school: Other students seem to like me. |  |  | Agreement: 1,2 |
|  | ST034Q06TA | Thinking about your school: I feel lonely at school. |  |  | Agreement: 1,2 |
|  | ST160Q01IA | How much do you agree or disagree? I read only if I have to. |  |  | Agreement: 3,4 |
|  | ST160Q02IA | How much do you agree or disagree? Reading is one of my favourite hobbies. |  |  | Agreement: 3,4 |
|  | ST160Q03IA | How much do you agree or disagree? I like talking about books with other people. |  |  | Agreement: 3,4 |
|  | ST160Q04IA | How much do you agree or disagree? For me, reading is a waste of time. |  |  | Agreement: 3,4 |
|  | ST160Q05IA | How much do you agree or disagree? I read only to get information that I need. |  |  | Agreement: 3,4 |
|  | ST161Q01HA | Agree: I am a good reader. |  |  | Agreement: 3,4 |
|  | ST161Q02HA | Agree: I am able to understand difficult texts. |  |  | Agreement: 3,4 |
|  | ST161Q03HA | Agree: I read fluently. |  |  | Agreement: 3,4 |
|  | ST161Q06HA | Agree: I have always had difficulty with reading. |  |  | Agreement: 3,4 |
|  | ST161Q07HA | Agree: I have to read a text several times before completely understanding it. |  |  | Agreement: 3,4 |
|  | ST161Q08HA | Agree: I find it difficult to answer questions about a text. |  |  | Agreement: 3,4 |
|  | ST185Q01HA | Agree: My life has clear meaning or purpose. |  |  | Agreement: 3,4 |
|  | ST185Q02HA | Agree: I have discovered a satisfactory meaning in life. |  |  | Agreement: 3,4 |
|  | ST185Q03HA | Agree: I have a clear sense of what gives meaning to my life. |  |  | Agreement: 3,4 |
|  | ST204Q02HA | Agree: Immigrant children should have the same opportunities for education that other children in the country have. |  |  | Agreement: 3,4 |
|  | ST204Q03HA | Agree: Immigrants who live in a country for several years should have the opportunity to vote in elections. |  |  | Agreement: 3,4 |
|  | ST204Q04HA | Agree: Immigrants should have the opportunity to continue their own customs and lifestyle. |  |  | Agreement: 3,4 |
|  | ST204Q05HA | Agree: Immigrants should have all the same rights that everyone else in the country has. |  |  | Agreement: 3,4 |
|  | ST207Q01HA | Agree: It irritates me when nobody defends bullied students. |  |  | Agreement: 3,4 |
|  | ST207Q02HA | Agree: It is a good thing to help students who can't defend themselves. |  |  | Agreement: 3,4 |
|  | ST207Q03HA | Agree: It is a wrong thing to join in bullying. |  |  | Agreement: 3,4 |
|  | ST207Q04HA | Agree: I feel bad seeing other students bullied. |  |  | Agreement: 3,4 |
|  | ST207Q05HA | Agree: I like it when someone stands up for other students who are being bullied. |  |  | Agreement: 3,4 |
| 2012 | ST29Q01 | Maths Interest - Enjoy Reading |  |  | Agreement: 1,2 |
|  | ST29Q02 | Instrumental Motivation - Worthwhile for Work |  |  | Agreement: 1,2 |
|  | ST29Q03 | Maths Interest - Look Forward to Lessons |  |  | Agreement: 1,2 |
|  | ST29Q04 | Maths Interest - Enjoy Maths |  |  | Agreement: 1,2 |
|  | ST29Q05 | Instrumental Motivation - Worthwhile for Career Chances |  |  | Agreement: 1,2 |
|  | ST29Q06 | Maths Interest - Interested |  |  | Agreement: 1,2 |
|  | ST29Q07 | Instrumental Motivation - Important for Future Study |  |  | Agreement: 1,2 |
|  | ST29Q08 | Instrumental Motivation - Helps to Get a Job |  |  | Agreement: 1,2 |
|  | ST42Q01 | Maths Anxiety - Worry That It Will Be Difficult |  |  | Agreement: 1,2 |
|  | ST42Q02 | Maths Self-Concept - Not Good at Maths |  |  | Agreement: 1,2 |
|  | ST42Q03 | Maths Anxiety - Get Very Tense |  |  | Agreement: 1,2 |
|  | ST42Q04 | Maths Self-Concept - Get Good <Grades> |  |  | Agreement: 1,2 |
|  | ST42Q05 | Maths Anxiety - Get Very Nervous |  |  | Agreement: 1,2 |
|  | ST42Q06 | Maths Self-Concept - Learn Quickly |  |  | Agreement: 1,2 |
|  | ST42Q07 | Maths Self-Concept - One of Best Subjects |  |  | Agreement: 1,2 |
|  | ST42Q08 | Maths Anxiety - Feel Helpless |  |  | Agreement: 1,2 |
|  | ST42Q09 | Maths Self-Concept - Understand Difficult Work |  |  | Agreement: 1,2 |
|  | ST42Q10 | Maths Anxiety - Worry About Getting Poor <Grades> |  |  | Agreement: 1,2 |
|  | ST86Q01 | Student-Teacher Relations - Get Along with Teachers |  |  | Agreement: 1,2 |
|  | ST86Q02 | Student-Teacher Relations - Teachers Are Interested |  |  | Agreement: 1,2 |
|  | ST86Q03 | Student-Teacher Relations - Teachers Listen to Students |  |  | Agreement: 1,2 |
|  | ST86Q04 | Student-Teacher Relations - Teachers Help Students |  |  | Agreement: 1,2 |
|  | ST86Q05 | Student-Teacher Relations - Teachers Treat Students Fair |  |  | Agreement: 1,2 |
|  | ST87Q01 | Sense of Belonging - Feel Like Outsider |  |  | Agreement: 1,2 |
|  | ST87Q02 | Sense of Belonging - Make Friends Easily |  |  | Agreement: 1,2 |
|  | ST87Q03 | Sense of Belonging - Belong at School |  |  | Agreement: 1,2 |
|  | ST87Q04 | Sense of Belonging - Feel Awkward at School |  |  | Agreement: 1,2 |
|  | ST87Q05 | Sense of Belonging - Liked by Other Students |  |  | Agreement: 1,2 |
|  | ST87Q06 | Sense of Belonging - Feel Lonely at School |  |  | Agreement: 1,2 |
|  | ST87Q07 | Sense of Belonging - Feel Happy at School |  |  | Agreement: 1,2 |
|  | ST87Q08 | Sense of Belonging - Things Are Ideal at School |  |  | Agreement: 1,2 |
|  | ST87Q09 | Sense of Belonging - Satisfied at School |  |  | Agreement: 1,2 |
|  | ST88Q01 | Attitude towards School - Does Little to Prepare Me for Life |  |  | Agreement: 1,2 |
|  | ST88Q02 | Attitude towards School - Waste of Time |  |  | Agreement: 1,2 |
|  | ST88Q03 | Attitude towards School - Gave Me Confidence |  |  | Agreement: 1,2 |
|  | ST88Q04 | Attitude towards School - Useful for Job |  |  | Agreement: 1,2 |
| 2000 | ST30Q01 | Well with students - Q30a |  |  | Agreement: 3,4 |
|  | ST30Q02 | Interested in students - Q30b |  |  | Agreement: 3,4 |
|  | ST30Q03 | Listen to me - Q30c |  |  | Agreement: 3,4 |
|  | ST30Q04 | Give extra help - Q30d |  |  | Agreement: 3,4 |
|  | ST30Q05 | Treat me fairly - Q30e |  |  | Agreement: 3,4 |
|  | ST31Q01 | Feel an outsider - Q31a |  |  | Agreement: 3,4 |
|  | ST31Q02 | Make friends - Q31b |  |  | Agreement: 3,4 |
|  | ST31Q03 | Feel I belong - Q31c |  |  | Agreement: 3,4 |
|  | ST31Q04 | Feel awkward - Q31d |  |  | Agreement: 3,4 |
|  | ST31Q05 | Think I'm liked - Q31e |  |  | Agreement: 3,4 |
|  | ST31Q06 | Feel lonely - Q31f |  |  | Agreement: 3,4 |
|  | ST31Q07 | Don't want to be - Q31g |  |  | Agreement: 3,4 |
|  | ST31Q08 | Feel Bored - Q31h |  |  | Agreement: 3,4 |
|  | ST35Q01 | Only if I have to - Q35a |  |  | Agreement: 3,4 |
|  | ST35Q02 | Favourite hobby - Q35b |  |  | Agreement: 3,4 |
|  | ST35Q03 | Talking about books - Q35c |  |  | Agreement: 3,4 |
|  | ST35Q04 | Hard to finish - Q35d |  |  | Agreement: 3,4 |
|  | ST35Q05 | Feel happy - Q35e |  |  | Agreement: 3,4 |
|  | ST35Q06 | Waste of time - Q35f |  |  | Agreement: 3,4 |
|  | ST35Q07 | Enjoy library - Q35g |  |  | Agreement: 3,4 |
|  | ST35Q08 | For information - Q35h |  |  | Agreement: 3,4 |
|  | ST35Q09 | Few minutes only - Q35i |  |  | Agreement: 3,4 |

*Note.* ERS = extreme response style; ARS = acquiescent response style. ERS was coded as the proportion of valid responses falling in either of the two endpoint categories of each four-category response scale. ARS was coded as the proportion of valid responses falling in the agreement categories. Student-level ERS and ARS indices were subsequently aggregated to the country level using the PISA final student weights.

**Table S13.** Multilevel Sensitivity Analyses of the Association Between Economic Inequality and Academic Perseverance After Adjusting for Extreme Response Style Across Four PISA Waves

| Study | Variable | *b* | *SE* | CI_lower | CI_upper | *p* |
| --- | --- | --- | --- | --- | --- | --- |
| PISA 2022 | GINI2020 | 0.006 | 0.005 | -0.003 | 0.015 | .208 |
|  | ESCS_cwc | 0.104 | 0.008 | 0.089 | 0.119 | < .001 |
|  | ESCS_cm | -0.21 | 0.094 | -0.394 | -0.026 | .032 |
|  | logGDP2020 | 0.1 | 0.103 | -0.102 | 0.302 | .340 |
|  | logPopulation2020 | -0.033 | 0.037 | -0.104 | 0.039 | .375 |
|  | Unemployment2020 | -0.001 | 0.006 | -0.012 | 0.010 | .865 |
|  | Gender | 0.091 | 0.004 | 0.084 | 0.099 | < .001 |
|  | Age | 0.042 | 0.006 | 0.029 | 0.054 | < .001 |
|  | Origin | 0.059 | 0.007 | 0.045 | 0.074 | < .001 |
|  | ERS | 0.98 | 0.459 | 0.081 | 1.879 | .040 |
|  | GINI2020:ESCS_cwc | -0.003 | 0.001 | -0.005 | -0.001 | .002 |
| PISA 2018 | GINI2016 | 0.007 | 0.003 | 0.002 | 0.013 | .014 |
|  | ESCS_cwc | 0.048 | 0.004 | 0.041 | 0.055 | < .001 |
|  | ESCS_cm | -0.059 | 0.051 | -0.158 | 0.040 | .248 |
|  | logGDP2016 | -0.118 | 0.061 | -0.238 | 0.001 | .058 |
|  | logPopulation2016 | -0.011 | 0.024 | -0.058 | 0.036 | .652 |
|  | Unemployment2016 | 0.001 | 0.003 | -0.005 | 0.008 | .715 |
|  | Gender | -0.076 | 0.002 | -0.079 | -0.073 | < .001 |
|  | Age | -0.033 | 0.003 | -0.039 | -0.027 | < .001 |
|  | Origin | 0.047 | 0.004 | 0.04 | 0.055 | < .001 |
|  | ERS | 0.589 | 0.336 | -0.07 | 1.248 | .085 |
|  | GINI2016:ESCS_cwc | -0.001 | 0.001 | -0.002 | -0.000 | .018 |
| PISA 2012 | GINI2010 | 0.013 | 0.006 | 0.002 | 0.024 | .029 |
|  | ESCS_cwc | 0.109 | 0.008 | 0.092 | 0.125 | < .001 |
|  | ESCS_cm | 0.069 | 0.104 | -0.134 | 0.273 | .506 |
|  | logGDP2010 | -0.411 | 0.125 | -0.657 | -0.165 | .002 |
|  | logPopulation2010 | 0.335 | 0.137 | 0.068 | 0.603 | .018 |
|  | Unemployment2010 | -0.007 | 0.007 | -0.021 | 0.007 | .350 |
|  | Gender | 0.025 | 0.004 | 0.017 | 0.032 | < .001 |
|  | Age | 0.057 | 0.007 | 0.044 | 0.070 | < .001 |
|  | Origin | -0.121 | 0.012 | -0.145 | -0.097 | < .001 |
|  | ERS | 0.405 | 0.615 | -0.801 | 1.612 | .513 |
|  | GINI2010:ESCS_cwc | -0.003 | 0.001 | -0.005 | -0.001 | .011 |
| PISA 2000 | GINI1998 | 0.018 | 0.005 | 0.008 | 0.028 | .009 |
|  | ESCS_cwc | 0.1 | 0.021 | 0.06 | 0.140 | < .001 |
|  | ESCS_cm | 0.167 | 0.134 | -0.095 | 0.430 | .246 |
|  | logGDP1998 | -0.238 | 0.187 | -0.606 | 0.129 | .236 |
|  | logPopulation1998 | -0.083 | 0.061 | -0.204 | 0.037 | .209 |
|  | Unemployment1998 | 0.019 | 0.011 | -0.003 | 0.040 | .120 |
|  | Gender | -0.248 | 0.01 | -0.268 | -0.228 | < .001 |
|  | Age | 0.008 | 0.002 | 0.005 | 0.011 | < .001 |
|  | Origin | 0.138 | 0.021 | 0.096 | 0.179 | < .001 |
|  | ERS | 0.906 | 0.534 | -0.14 | 1.953 | .127 |
|  | GINI1998:ESCS_cwc | -0.003 | 0.002 | -0.007 | 0.001 | .194 |

*Note.* Unstandardized regression coefficients are reported. CI = confidence interval; ESCS = index of economic, social, and cultural status; ESCS_cwc = country-mean-centered student-level ESCS; ESCS_cm = country mean ESCS; ERS = extreme response style. Gini coefficients and continuous covariates were grand-mean centered, whereas ESCS was centered within countries. All models included student sampling weights, random intercepts for schools and countries, and a random slope for ESCS_cwc across countries. The Gini × ESCS_cwc term represents the cross-level interaction between country-level economic inequality and individual-level family socioeconomic status. ERS was included as a country-level response-style covariate. Models were estimated separately for PISA 2000, 2012, 2018, and 2022.

**Table S14.** Multilevel Sensitivity Analyses of the Association Between Economic Inequality and Academic Perseverance After Adjusting for Acquiescent Response Style Across Four PISA Waves

| Study | Variable | *b* | *SE* | CI_lower | CI_upper | *p* |
| --- | --- | --- | --- | --- | --- | --- |
| PISA 2022 | GINI2020 | 0.008 | 0.006 | -0.004 | 0.019 | .209 |
|  | ESCS_cwc | 0.104 | 0.008 | 0.089 | 0.119 | < .001 |
|  | ESCS_cm | -0.204 | 0.101 | -0.401 | -0.007 | .051 |
|  | logGDP2020 | 0.166 | 0.104 | -0.038 | 0.370 | .121 |
|  | logPopulation2020 | -0.042 | 0.039 | -0.12 | 0.035 | .287 |
|  | Unemployment2020 | 0 | 0.006 | -0.012 | 0.012 | .966 |
|  | Gender | 0.091 | 0.004 | 0.084 | 0.099 | < .001 |
|  | Age | 0.042 | 0.006 | 0.029 | 0.054 | < .001 |
|  | Origin | 0.06 | 0.007 | 0.045 | 0.074 | < .001 |
|  | ARS | 0.418 | 0.858 | -1.263 | 2.099 | .629 |
|  | GINI2020:ESCS_cwc | -0.003 | 0.001 | -0.005 | -0.001 | .002 |
| PISA 2018 | GINI2016 | 0.008 | 0.003 | 0.003 | 0.013 | .003 |
|  | ESCS_cwc | 0.048 | 0.004 | 0.041 | 0.055 | < .001 |
|  | ESCS_cm | -0.046 | 0.047 | -0.139 | 0.047 | .336 |
|  | logGDP2016 | -0.037 | 0.051 | -0.138 | 0.063 | .471 |
|  | logPopulation2016 | -0.016 | 0.022 | -0.058 | 0.026 | .459 |
|  | Unemployment2016 | 0.002 | 0.003 | -0.003 | 0.008 | .433 |
|  | Gender | -0.076 | 0.002 | -0.079 | -0.073 | < .001 |
|  | Age | -0.033 | 0.003 | -0.039 | -0.027 | < .001 |
|  | Origin | 0.047 | 0.004 | 0.04 | 0.055 | < .001 |
|  | ARS | 1.139 | 0.323 | 0.505 | 1.772 | < .001 |
|  | GINI2016:ESCS_cwc | -0.001 | 0.001 | -0.002 | -0.000 | .018 |
| PISA 2012 | GINI2010 | 0.012 | 0.005 | 0.001 | 0.022 | .033 |
|  | ESCS_cwc | 0.109 | 0.008 | 0.092 | 0.125 | < .001 |
|  | ESCS_cm | 0.143 | 0.101 | -0.055 | 0.340 | .163 |
|  | logGDP2010 | -0.366 | 0.123 | -0.608 | -0.124 | .005 |
|  | logPopulation2010 | 0.299 | 0.13 | 0.045 | 0.554 | .026 |
|  | Unemployment2010 | -0.003 | 0.007 | -0.017 | 0.011 | .667 |
|  | Gender | 0.025 | 0.004 | 0.017 | 0.032 | < .001 |
|  | Age | 0.057 | 0.007 | 0.044 | 0.070 | < .001 |
|  | Origin | -0.121 | 0.012 | -0.145 | -0.097 | < .001 |
|  | ARS | 1.222 | 0.564 | 0.117 | 2.327 | .036 |
|  | GINI2010:ESCS_cwc | -0.003 | 0.001 | -0.005 | -0.001 | .011 |
| PISA 2000 | GINI1998 | 0.022 | 0.005 | 0.012 | 0.032 | .002 |
|  | ESCS_cwc | 0.1 | 0.02 | 0.06 | 0.139 | < .001 |
|  | ESCS_cm | 0.144 | 0.167 | -0.183 | 0.471 | .409 |
|  | logGDP1998 | -0.148 | 0.201 | -0.542 | 0.246 | .479 |
|  | logPopulation1998 | -0.09 | 0.069 | -0.225 | 0.045 | .222 |
|  | Unemployment1998 | 0.02 | 0.012 | -0.003 | 0.044 | .119 |
|  | Gender | -0.248 | 0.01 | -0.268 | -0.228 | < .001 |
|  | Age | 0.008 | 0.002 | 0.005 | 0.010 | < .001 |
|  | Origin | 0.139 | 0.021 | 0.097 | 0.180 | < .001 |
|  | ARS | -1.075 | 1.648 | -4.304 | 2.155 | .530 |
|  | GINI1998:ESCS_cwc | -0.003 | 0.002 | -0.007 | 0.001 | .189 |

*Note.* Unstandardized regression coefficients are reported. CI = confidence interval; ESCS = index of economic, social, and cultural status; ESCS_cwc = country-mean-centered student-level ESCS; ESCS_cm = country mean ESCS; ARS = acquiescent response style. Gini coefficients and continuous covariates were grand-mean centered, whereas ESCS was centered within countries. All models included student sampling weights, random intercepts for schools and countries, and a random slope for ESCS_cwc across countries. The Gini × ESCS_cwc term represents the cross-level interaction between country-level economic inequality and individual-level family socioeconomic status. ARS was included as a country-level response-style covariate. Models were estimated separately for PISA 2000, 2012, 2018, and 2022.

**Table S15.** Sensitivity Analyses of the Country-Level Association Between Economic Inequality and Academic Perseverance

| Study | *β*_Baseline_ | *p*_Baseline_ | *β*_LOCO_ range | Worst-case *p*_LOCO_ | *β*_Excluding Latin America_ | *p*_Excluding Latin America_ | *β*_region FE_ | *p*_region FE_ |
| --- | --- | --- | --- | --- | --- | --- | --- | --- |
| 2022 | 0.014 | .001 | [.012, .016] | .003 | 0.012 | .007 | 0.016 | .022 |
| 2018 | 0.010 | <.001 | [.009, .010] | <.001 | 0.008 | .006 | 0.003 | .329 |
| 2012 | 0.012 | .019 | [.010, .016] | .042 | 0.012 | .047 | 0.027 | .002 |
| 2000 | 0.015 | .002 | [.011, .018] | .023 | 0.011 | .023 | 0.019 | .044 |

*Note.* Baseline estimates are country-level regression coefficients for the association between the Gini coefficient and national academic perseverance, adjusting for GDP, population, and unemployment. LOCO = leave-one-country-out analysis. The LOCO β range represents the minimum and maximum Gini coefficients obtained after sequentially excluding each country. The worst-case LOCO *p* value is the largest *p* value observed across all leave-one-country-out models. “Excluding Latin America” reports estimates obtained after removing countries in the Latin America and Caribbean region. “region FE” reports estimates from models additionally including fixed effects for a predefined intermediate-granularity world-region classification.

**Table S16.** Sensitivity Analyses of the Gini Coefficient and Gini × ESCS Interaction Across Alternative Lag Specifications

| Study | spec | Variable | n_countries | *b* | *SE* | CI_lower | CI_upper | *p* |
| --- | --- | --- | --- | --- | --- | --- | --- | --- |
| PISA2022 | lag0 | Gini main effect | 37 | 0.011 | 0.004 | 0.002 | 0.020 | .022 |
|  |  | Gini × ESCS | 37 | -0.004 | 0.001 | -0.006 | -0.001 | .002 |
|  | lag1 | Gini main effect | 37 | 0.008 | 0.004 | 0.000 | 0.017 | .053 |
|  |  | Gini × ESCS | 37 | -0.003 | 0.001 | -0.006 | -0.001 | .003 |
|  | lag2 | Gini main effect | 37 | 0.009 | 0.004 | 0.000 | 0.018 | .040 |
|  |  | Gini × ESCS | 37 | -0.003 | 0.001 | -0.005 | -0.001 | .001 |
|  | lag3 | Gini main effect | 37 | 0.007 | 0.004 | -0.002 | 0.016 | .129 |
|  |  | Gini × ESCS | 37 | -0.004 | 0.001 | -0.006 | -0.002 | .001 |
|  | lag4 | Gini main effect | 37 | 0.007 | 0.005 | -0.002 | 0.017 | .133 |
|  |  | Gini × ESCS | 37 | -0.004 | 0.001 | -0.006 | -0.001 | .002 |
|  | lag5 | Gini main effect | 37 | 0.010 | 0.004 | 0.002 | 0.019 | .020 |
|  |  | Gini × ESCS | 37 | -0.004 | 0.001 | -0.006 | -0.002 | .001 |
|  | lag6 | Gini main effect | 37 | 0.009 | 0.004 | 0.000 | 0.018 | .043 |
|  |  | Gini × ESCS | 37 | -0.004 | 0.001 | -0.006 | -0.002 | .001 |
|  | lag7 | Gini main effect | 37 | 0.011 | 0.005 | 0.002 | 0.021 | .023 |
|  |  | Gini × ESCS | 37 | -0.004 | 0.001 | -0.006 | -0.002 | .001 |
|  | lag8 | Gini main effect | 37 | 0.010 | 0.004 | 0.001 | 0.019 | .030 |
|  |  | Gini × ESCS | 37 | -0.004 | 0.001 | -0.006 | -0.002 | .001 |
|  | lag9 | Gini main effect | 37 | 0.010 | 0.004 | 0.001 | 0.020 | .025 |
|  |  | Gini × ESCS | 37 | -0.004 | 0.001 | -0.006 | -0.001 | .002 |
|  | lag10 | Gini main effect | 37 | 0.011 | 0.004 | 0.002 | 0.020 | .019 |
|  |  | Gini × ESCS | 37 | -0.004 | 0.001 | -0.006 | -0.001 | .002 |
|  | avg_lag1_3 | Gini main effect | 37 | 0.008 | 0.004 | 0.000 | 0.017 | .061 |
|  |  | Gini × ESCS | 37 | -0.004 | 0.001 | -0.006 | -0.001 | .002 |
|  | avg_lag1_5 | Gini main effect | 37 | 0.009 | 0.004 | 0.000 | 0.018 | .054 |
|  |  | Gini × ESCS | 37 | -0.004 | 0.001 | -0.006 | -0.001 | .002 |
|  | avg_lag1_10 | Gini main effect | 37 | 0.010 | 0.005 | 0.001 | 0.020 | .033 |
|  |  | Gini × ESCS | 37 | -0.004 | 0.001 | -0.006 | -0.002 | .002 |
| B_PISA2018 | lag0 | Gini main effect | 67 | 0.007 | 0.003 | 0.001 | 0.013 | .019 |
|  |  | Gini × ESCS | 67 | -0.001 | 0.001 | -0.002 | 0.000 | .025 |
|  | lag1 | Gini main effect | 67 | 0.009 | 0.003 | 0.003 | 0.014 | .003 |
|  |  | Gini × ESCS | 67 | -0.001 | 0.001 | -0.002 | 0.000 | .013 |
|  | lag2 | Gini main effect | 67 | 0.009 | 0.003 | 0.003 | 0.014 | .002 |
|  |  | Gini × ESCS | 67 | -0.001 | 0.001 | -0.002 | 0.000 | .019 |
|  | lag3 | Gini main effect | 67 | 0.008 | 0.003 | 0.003 | 0.014 | .005 |
|  |  | Gini × ESCS | 67 | -0.001 | 0.001 | -0.002 | 0.000 | .012 |
|  | lag4 | Gini main effect | 67 | 0.008 | 0.003 | 0.003 | 0.014 | .004 |
|  |  | Gini × ESCS | 67 | -0.001 | 0.001 | -0.002 | 0.000 | .013 |
|  | lag5 | Gini main effect | 67 | 0.007 | 0.003 | 0.002 | 0.012 | .012 |
|  |  | Gini × ESCS | 67 | -0.001 | 0.001 | -0.002 | 0.000 | .027 |
|  | lag6 | Gini main effect | 66 | 0.007 | 0.003 | 0.002 | 0.012 | .010 |
|  |  | Gini × ESCS | 66 | -0.001 | 0.000 | -0.002 | 0.000 | .022 |
|  | lag7 | Gini main effect | 66 | 0.008 | 0.003 | 0.003 | 0.014 | .002 |
|  |  | Gini × ESCS | 66 | -0.001 | 0.000 | -0.002 | 0.000 | .019 |
|  | lag8 | Gini main effect | 65 | 0.008 | 0.003 | 0.003 | 0.013 | .002 |
|  |  | Gini × ESCS | 65 | -0.001 | 0.000 | -0.002 | 0.000 | .032 |
|  | lag9 | Gini main effect | 65 | 0.008 | 0.002 | 0.003 | 0.013 | .001 |
|  |  | Gini × ESCS | 65 | -0.001 | 0.000 | -0.002 | 0.000 | .034 |
|  | lag10 | Gini main effect | 65 | 0.007 | 0.002 | 0.002 | 0.012 | .003 |
|  |  | Gini × ESCS | 65 | -0.001 | 0.000 | -0.002 | 0.000 | .046 |
|  | avg_lag1_3 | Gini main effect | 67 | 0.009 | 0.003 | 0.003 | 0.014 | .003 |
|  |  | Gini × ESCS | 67 | -0.001 | 0.001 | -0.002 | 0.000 | .014 |
|  | avg_lag1_5 | Gini main effect | 67 | 0.008 | 0.003 | 0.003 | 0.014 | .004 |
|  |  | Gini × ESCS | 67 | -0.001 | 0.001 | -0.002 | 0.000 | .016 |
|  | avg_lag1_10 | Gini main effect | 67 | 0.009 | 0.003 | 0.003 | 0.014 | .002 |
|  |  | Gini × ESCS | 67 | -0.001 | 0.001 | -0.002 | 0.000 | .023 |
| C_PISA2012 | lag0 | Gini main effect | 50 | 0.017 | 0.006 | 0.006 | 0.028 | .004 |
|  |  | Gini × ESCS | 50 | -0.003 | 0.001 | -0.005 | -0.001 | .018 |
|  | lag1 | Gini main effect | 50 | 0.017 | 0.006 | 0.006 | 0.029 | .004 |
|  |  | Gini × ESCS | 50 | -0.003 | 0.001 | -0.006 | -0.001 | .009 |
|  | lag2 | Gini main effect | 50 | 0.015 | 0.005 | 0.004 | 0.026 | .008 |
|  |  | Gini × ESCS | 50 | -0.003 | 0.001 | -0.005 | -0.001 | .011 |
|  | lag3 | Gini main effect | 50 | 0.013 | 0.005 | 0.003 | 0.023 | .014 |
|  |  | Gini × ESCS | 50 | -0.003 | 0.001 | -0.005 | -0.001 | .016 |
|  | lag4 | Gini main effect | 50 | 0.012 | 0.005 | 0.001 | 0.022 | .025 |
|  |  | Gini × ESCS | 50 | -0.003 | 0.001 | -0.005 | -0.001 | .015 |
|  | lag5 | Gini main effect | 49 | 0.013 | 0.005 | 0.003 | 0.022 | .010 |
|  |  | Gini × ESCS | 49 | -0.003 | 0.001 | -0.005 | 0.000 | .019 |
|  | lag6 | Gini main effect | 49 | 0.013 | 0.005 | 0.004 | 0.022 | .007 |
|  |  | Gini × ESCS | 49 | -0.003 | 0.001 | -0.005 | 0.000 | .023 |
|  | lag7 | Gini main effect | 48 | 0.012 | 0.004 | 0.003 | 0.021 | .011 |
|  |  | Gini × ESCS | 48 | -0.002 | 0.001 | -0.004 | 0.000 | .073 |
|  | lag8 | Gini main effect | 48 | 0.010 | 0.005 | 0.001 | 0.019 | .038 |
|  |  | Gini × ESCS | 48 | -0.003 | 0.001 | -0.005 | 0.000 | .023 |
|  | lag9 | Gini main effect | 48 | 0.011 | 0.004 | 0.002 | 0.019 | .016 |
|  |  | Gini × ESCS | 48 | -0.002 | 0.001 | -0.004 | 0.000 | .030 |
|  | lag10 | Gini main effect | 46 | 0.010 | 0.004 | 0.002 | 0.018 | .018 |
|  |  | Gini × ESCS | 46 | -0.002 | 0.001 | -0.004 | 0.000 | .032 |
|  | avg_lag1_3 | Gini main effect | 50 | 0.015 | 0.005 | 0.004 | 0.026 | .008 |
|  |  | Gini × ESCS | 50 | -0.003 | 0.001 | -0.005 | -0.001 | .012 |
|  | avg_lag1_5 | Gini main effect | 50 | 0.014 | 0.005 | 0.003 | 0.025 | .010 |
|  |  | Gini × ESCS | 50 | -0.003 | 0.001 | -0.005 | -0.001 | .014 |
|  | avg_lag1_10 | Gini main effect | 50 | 0.013 | 0.005 | 0.003 | 0.023 | .012 |
|  |  | Gini × ESCS | 50 | -0.003 | 0.001 | -0.005 | 0.000 | .021 |
| D_PISA2000 | lag0 | Gini main effect | 22 | 0.024 | 0.005 | 0.012 | 0.035 | .001 |
|  |  | Gini × ESCS | 22 | -0.002 | 0.002 | -0.005 | 0.002 | .289 |
|  | lag1 | Gini main effect | 22 | 0.021 | 0.005 | 0.010 | 0.031 | .001 |
|  |  | Gini × ESCS | 22 | -0.002 | 0.002 | -0.005 | 0.002 | .271 |
|  | lag2 | Gini main effect | 22 | 0.020 | 0.005 | 0.010 | 0.030 | .001 |
|  |  | Gini × ESCS | 22 | -0.002 | 0.002 | -0.005 | 0.001 | .249 |
|  | lag3 | Gini main effect | 22 | 0.020 | 0.005 | 0.009 | 0.031 | .002 |
|  |  | Gini × ESCS | 22 | -0.002 | 0.002 | -0.005 | 0.002 | .289 |
|  | lag4 | Gini main effect | 22 | 0.021 | 0.005 | 0.010 | 0.032 | .001 |
|  |  | Gini × ESCS | 22 | -0.002 | 0.002 | -0.005 | 0.002 | .303 |
|  | lag5 | Gini main effect | 21 | 0.023 | 0.005 | 0.013 | 0.033 | <.001 |
|  |  | Gini × ESCS | 21 | -0.002 | 0.002 | -0.005 | 0.002 | .310 |
|  | lag6 | Gini main effect | 20 | 0.023 | 0.005 | 0.013 | 0.033 | <.001 |
|  |  | Gini × ESCS | 20 | -0.001 | 0.002 | -0.005 | 0.002 | .380 |
|  | lag7 | Gini main effect | 18 | 0.025 | 0.006 | 0.013 | 0.038 | .001 |
|  |  | Gini × ESCS | 18 | -0.002 | 0.002 | -0.005 | 0.002 | .291 |
|  | lag8 | Gini main effect | 18 | 0.024 | 0.005 | 0.012 | 0.036 | .001 |
|  |  | Gini × ESCS | 18 | -0.002 | 0.002 | -0.006 | 0.002 | .253 |
|  | lag9 | Gini main effect | 11 | 0.027 | 0.007 | 0.008 | 0.046 | .017 |
|  |  | Gini × ESCS | 11 | -0.002 | 0.002 | -0.007 | 0.003 | .362 |
|  | lag10 | Gini main effect | 7 | -0.014 | 0.022 | -0.167 | 0.140 | .619 |
|  |  | Gini × ESCS | 7 | -0.003 | 0.004 | -0.012 | 0.007 | .476 |
|  | avg_lag1_3 | Gini main effect | 22 | 0.020 | 0.005 | 0.010 | 0.031 | .001 |
|  |  | Gini × ESCS | 22 | -0.002 | 0.002 | -0.005 | 0.002 | .269 |
|  | avg_lag1_5 | Gini main effect | 22 | 0.021 | 0.005 | 0.010 | 0.031 | .001 |
|  |  | Gini × ESCS | 22 | -0.002 | 0.002 | -0.005 | 0.002 | .276 |
|  | avg_lag1_10 | Gini main effect | 22 | 0.021 | 0.005 | 0.010 | 0.032 | .001 |
|  |  | Gini × ESCS | 22 | -0.002 | 0.002 | -0.005 | 0.001 | .263 |

Note. Gini estimates were examined using contemporaneous values (lag 0), values lagged by 1–10 years, and averages over the preceding 1–3, 1–5, and 1–10 years. All models included the same covariates as the corresponding main analyses and were estimated using multilevel models with students nested within schools and countries. *b* = unstandardized regression coefficient; *SE* = standard error; CI = confidence interval; ESCS = index of economic, social, and cultural status.

# Section S8. PISA Data Exclusion Criteria

## PISA 2022

### Countries/Regions Excluded Due to Missing GINI 2020 or Earlier Data (N = ****59,578****):

### **Brunei Darussalam** (N=5,576), **Cambodia** (N=5,279), **Chinese Taipei** (N=5,857), **Hong Kong (China)** (N=5,907), **Korea** (N=6,454), **Macao (China)** (N=4,384), **New Zealand** (N=4,682), **Palestinian Authority** (N=7,905), **Saudi Arabia** (N=6,928), **Singapore** (N=6,606).

### Participants Excluded Due to Missing Perseverance Responses: ****N = 239,691.****

## PISA 2018

### Countries/Regions Excluded Due to Missing GINI 2016 or Earlier Data (N = ****50,696****):

**Brunei Darussalam** (N=6,828), **Hong Kong (China)** (N=6,037)**, Macao (China)** (N=3,775), **New Zealand** (N=6,173), **Qatar** (N=13,828), **Saudi Arabia** (N=6,136), **Singapore** (N=6,676), **Chinese Taipei** (N=7,243).

### Participants Excluded Due to Missing Perseverance Responses: ****N = 60,749.****

## PISA 2012

### Countries/Regions Excluded Due to Missing GINI 2012 or Earlier Data (N = ****64,684)****:

### **Chinese Taipei** (N=6,046), **Hong Kong (China)** (N=4,670), **Liechtenstein** (N=293), **Macao (China)** (N=5,335), **Montenegro** (N=4,744), **New Zealand** (N=4,291), **Qatar** (N=10,966), **Republic of Serbia** (N=4,684), **Russian Federation** (N=1,761), **Saudi Arabia** (N=11,500), **Singapore** (N=5,546), **Turkey** (N=4,848).

### Participants Excluded Due to Missing Perseverance Responses: ****N = 145,525.****

## PISA 2000

### Countries/Regions Excluded Due to Missing GINI 1998 or Earlier Data (N = ****34,264)****:

### **Bulgaria** (N=2,614), **Hong Kong (China)** (N=2,438), **Hungary** (N=2,799), **Iceland** (N=1,882), **Japan** (N=2,924), **Korea** (N=2,769), **Latvia** (N=2,149), **Liechtenstein** (N=175), **Macedonia** (N=2,544), **New Zealand** (N=2,048), **Poland** (N=1,976), **Portugal** (N=2,545), **Romania** (N=2,682), **Russian Federation** (N=3,719).

### Participants Excluded Due to Missing Perseverance Responses:****N = 41,613.****

## Section S9. Included in the analysis of the national situation report

### Participation in each year

### PISA 2022 (N=44)

Argentina, Australia, Austria, Belgium, Brazil, Bulgaria, Canada, Chile, Colombia, Costa Rica, Croatia, Czech Republic, Denmark, Estonia, Finland, France, Germany, Greece, Hungary, Iceland, Ireland, Italy, Jamaica, Latvia, Lithuania, Malta, Mexico, Republic of Moldova, Montenegro, Netherlands, Norway, Peru, Poland, Portugal, Romania, Serbia, Slovak Republic, Slovenia, Spain, Switzerland, Türkiye, Ukrainian regions (18 of 27), United Kingdom, Uruguay

### PISA 2018 (N=72)

Albania, United Arab Emirates, Argentina, Australia, Austria, Belgium, Bulgaria, Bosnia and Herzegovina, Belarus, Brazil, Canada, Switzerland, Chile, Colombia, Costa Rica, Czechia, Germany, Denmark, Dominican Republic, Spain, Estonia, Finland, France, United Kingdom, Georgia, Greece, Croatia, Hungary, India, Ireland, Iceland, Israel, Italy, Jordan, Japan, Kazakhstan, Korea, Kosovo, Lebanon, Lithuania, Luxembourg, Latvia, Morocco, Moldova, Mexico, North Macedonia, Malta, Montenegro, Malaysia, Netherlands, Norway, Panama, Peru, Philippines, Poland, Portugal, Azerbaijan, China, Moscow Region (RUS), Tatarstan (RUS), Romania, Russian Federation, Serbia, Slovak Republic, Slovenia, Sweden, Thailand, Turkey, Ukraine, Uruguay, United States, Vietnam

### PISA 2012 (N=52)

Albania, Argentina, Australia, Austria, Belgium, Bulgaria, Brazil, Canada, Switzerland, Chile, Colombia, Costa Rica, Czech Republic, Germany, Denmark, Spain, Estonia, Finland, France, United Kingdom, Greece, Croatia, Hungary, Indonesia, Ireland, Iceland, Israel, Italy, Jordan, Japan, Kazakhstan, Korea, Lithuania, Luxembourg, Latvia, Mexico, Malaysia, Netherlands, Norway, Peru, Poland, Portugal, China, Romania, Slovak Republic, Slovenia, Sweden, Thailand, Tunisia, Uruguay, United States, Vietnam

### PISA 2000 (N=22)

Albania, Australia, Austria, Belgium, Brazil, Switzerland, Chile, Czech Republic, Germany, Denmark, Finland, United Kingdom, Ireland, Israel, Italy, Luxembourg, Mexico, Netherlands, Norway, Sweden, Thailand, United States

**Complete list of countries/regions (N=73, sorted alphabetically) :**

1. Albania
2. Argentina
3. Australia
4. Austria
5. Azerbaijan
6. Belarus
7. Belgium
8. Bosnia and Herzegovina
9. Brazil
10. Bulgaria
11. Canada
12. Chile
13. China
14. Colombia
15. Costa Rica
16. Croatia
17. Czech Republic
18. Denmark
19. Dominican Republic
20. Estonia
21. Finland
22. France
23. Georgia
24. Germany
25. Greece
26. Hungary
27. Iceland
28. India
29. Indonesia
30. Ireland
31. Israel
32. Italy
33. Jamaica
34. Japan
35. Jordan
36. Kazakhstan
37. Kosovo
38. Latvia
39. Lebanon
40. Lithuania
41. Luxembourg
42. Malaysia
43. Malta
44. Mexico
45. Moldova
46. Montenegro
47. Morocco
48. Netherlands
49. North Macedonia
50. Norway
51. Panama
52. Peru
53. Philippines
54. Poland
55. Portugal
56. Romania
57. Russia
58. Serbia
59. Slovak Republic
60. Slovenia
61. South Korea
62. Spain
63. Sweden
64. Switzerland
65. Thailand
66. Tunisia
67. Türkiye
68. Ukraine
69. United Arab Emirates
70. United Kingdom
71. United States
72. Uruguay
73. Vietnam

**Section S10 Rationale for covariate inclusion**

**Gender.** Research indicates that women perceive economic inequality more strongly than men (Chen et al., 2024; Zamberlan et al., 2025), and that higher levels of economic inequality reinforce competitive normative climates that further constrain women's access to opportunities and resources (Moreno-Bella et al., 2022). Additionally, several empirical studies have found that girls demonstrate significantly higher levels of grit and perseverance than boys (Oriol et al., 2017; Kannangara et al., 2018; Christensen & Knezek, 2014). Given that gender is potentially associated with both perceived economic inequality and perseverance, it was included as a control variable in the analyses.

**Age.** Credé et al. (2017), in a meta-analytic synthesis of the grit literature encompassing 584 effect sizes from 88 independent samples (N = 66,807), established perseverance of effort as the core facet of grit and confirmed a positive association between grit and age. Shubert et al. (2020), using an accelerated longitudinal design with 4,055 adolescents in grades 6–12, further demonstrated that perseverance followed a growth trajectory in early-to-middle adolescence that leveled off in later adolescence. Together, these findings suggest that perseverance is developmentally sensitive during adolescence. Given that even modest age variation within the PISA sample may introduce systematic variance in perseverance scores, age was included as a covariate in the analyses.

**Immigrant Background.** Socioeconomic disadvantage is considerably more prevalent among immigrant students than among native students in most participating countries (OECD, 2023), suggesting that immigrant background may be associated with greater objective exposure to economic inequality. At the same time, a number of studies tentatively suggest that immigrant status may be linked to stronger academic perseverance. Fuligni (1997), drawing on a sample of approximately 1,100 adolescents from Latino, East Asian, Filipino, and European backgrounds, found that first- and second-generation immigrant students outperformed their native peers in mathematics and English, an advantage attributable in part to more positive academic attitudes and higher behavioral engagement. Chiu et al. (2012), in a multilevel analysis of 276,165 fifteen-year-olds across 41 countries, similarly found that immigrant students displayed more positive attitudes toward school than their native counterparts. Given that cognitive engagement with school overlaps conceptually with perseverance to some degree (Fredricks et al., 2004), these patterns may indirectly suggest a tendency toward greater sustained effort among immigrant students when facing academic challenges. In light of these considerations, immigrant background was included as a control variable in the analyses.

**Section S11. Power analysis procedures**

The effect size threshold (*f*² = .02) follows Cohen's (1988) convention for a small effect in multiple regression. The significance level was set at α = .05, consistent with standard practice in psychological research. We set a relatively stringent power level of *β* = .90 for each predictor in the regression model (Cohen, 1988). Following Sommet et al. (2023), given that the model includes 8 predictors, the overall required power was calculated as .90*^*(1/8) ≈ .987 to avoid Type II errors, and the overall power level was therefore set at *β* = .99. Power analyses were conducted in R (Version 4.2.3; R Core Team, 2023) using the pwr package (Version 1.3.0; Champely, 2020).

**References**

Champely, S. (2020). pwr: Basic functions for power analysis. R package version 1.3-0. <https://CRAN.R-project.org/package=pwr>

Chen, J. C., Yu, C., & Zhu, J. (2024). Gender differences in whether and how perceived inequality hampers self-rated health and mental health: Evidence from the Chinese General Social Survey and a randomized experiment in China. *International Journal of Environmental Research and Public Health*, *21*(12), Article 1640. <https://doi.org/10.3390/ijerph21121640>

Chiu, M. M., Pong, S., Mori, I., & Chow, B. W.-Y. (2012). Immigrant students' emotional and cognitive engagement at school: A multilevel analysis of students in 41 countries. *Journal of Youth and Adolescence*, *41*(11), 1409–1425. <https://doi.org/10.1007/s10964-012-9763-x>

Christensen, R., & Knezek, G. (2014). Comparative measures of grit, tenacity and perseverance. International Journal of Learning, *Teaching and Educational Research*, 8(1), 16–30.

Cohen, J. (1988). *Statistical power analysis for the behavioral sciences* (2nd ed.). Lawrence Erlbaum Associates.

Credé, M., Tynan, M. C., & Harms, P. D. (2017). Much ado about grit: A meta-analytic synthesis of the grit literature. *Journal of Personality and Social Psychology*, *113*(3), 492–511. <https://doi.org/10.1037/pspp0000102>

Fredricks, J. A., Blumenfeld, P. C., & Paris, A. H. (2004). School engagement: Potential of the concept, state of the evidence. *Review of Educational Research*, *74*(1), 59–109. <https://doi.org/10.3102/00346543074001059>

Fuligni, A. J. (1997). The academic achievement of adolescents from immigrant families: The roles of family background, attitudes, and behavior. *Child Development*, *68*(2), 351–363. <https://doi.org/10.1111/j.1467-8624.1997.tb01944.x>

Kannangara, C. S., Allen, R. E., Waugh, G., Nahar, N., Khan, S. Z. N., Rogerson, S., & Carson, J. (2018). All that glitters is not grit: Three studies of grit in university students. *Frontiers in Psychology*, *9*, Article 1539. <https://doi.org/10.3389/fpsyg.2018.01539>

Moreno-Bella, E., Kulich, C., Willis, G. B., & Moya, M. (2022). What about diversity? The effect of organizational economic inequality on the perceived presence of women and ethnic minority groups. *PLoS ONE*, *17*(8), Article e0271356. <https://doi.org/10.1371/journal.pone.0271356>

OECD. (2023). PISA 2022 results (Volume I): The state of learning and equity in education. OECD Publishing. <https://doi.org/10.1787/53f23881-en>

Oriol, X., Miranda, R., Oyanedel, J. C., & Torres, J. (2017). The role of self-control and grit in domains of school success in students of primary and secondary school. *Frontiers in Psychology*, *8*, 1716. [https://doi.org/](https://doi.org/) 10.3389/fpsyg.2017.01716

R Core Team. (2023). R: A language and environment for statistical computing. R Foundation for Statistical Computing. <https://www.R-project.org/>

Shubert, J., Wray-Lake, L., & McKay, B. (2020). Looking ahead and working hard: How school experiences foster adolescents' future orientation and perseverance. *Journal of Research on Adolescence*, *30*, 989–1007. <https://doi.org/10.1111/jora.12574>

Sommet, N., Weissman, D. L., & Elliot, A. J. (2023). Income inequality predicts competitiveness and cooperativeness at school. *Journal of Educational Psychology*, *115*(1), 173–191. https://doi.org/10.1037/edu0000731

Zamberlan, A., Galos, D. R., Strauß, S., & Hinz, T. (2025). Fairness Evaluations of Higher Education Graduates’ Earnings: The Role of Female Preference for Equality and Self-Interest. *The British Journal of Sociology*, *76*(3), 541–552. https://doi.org/10.1111/1468-4446.13192
